# A Walnut Diet in Combination with Enriched Environment Improves Cognitive Function and Affects Lipid Metabolites in Brain and Liver of Aged NMRI Mice

**DOI:** 10.1007/s12017-020-08639-7

**Published:** 2020-12-26

**Authors:** Carsten Esselun, Benjamin Dilberger, Carmina V. Silaidos, Elisabeth Koch, Nils Helge Schebb, Gunter P. Eckert

**Affiliations:** 1 Laboratory for Nutrition in Prevention and Therapy, Institute of Nutritional Sciences, Justus-Liebig-University, Biomedical Research Center Seltersberg (BFS), Schubertstr. 81, 35392 Giessen, Germany; 2 Chair of Food Chemistry, Faculty of Mathematics and Natural Sciences, University of Wuppertal, Gaussstr. 20, 42119 Wuppertal, Germany

**Keywords:** Mitochondrial function, Brain, Liver, Oxylipins, Cognition, Behaviour, NMRI, Ageing, Neurodegeneration

## Abstract

This in vivo study aimed to test if a diet enriched with 6% walnuts alone or in combination with physical activity supports healthy ageing by changing the oxylipin profile in brain and liver, improving motor function, cognition, and cerebral mitochondrial function. Female NMRI mice were fed a 6% walnut diet starting at an age of 12 months for 24 weeks. One group was additionally maintained in an enriched environment, one group without intervention served as control. After three months, one additional control group of young mice (3 weeks old) was introduced. Motor and cognitive functions were measured using Open Field, Y-Maze, Rotarod and Passive Avoidance tests. Lipid metabolite profiles were determined using RP-LC-ESI(-)-MS/MS in brain and liver tissues of mice. Cerebral mitochondrial function was characterized by the determination of ATP levels, mitochondrial membrane potential and mitochondrial respiration. Expression of genes involved with mito- and neurogenesis, inflammation, and synaptic plasticity were determined using qRT-PCR. A 6% walnut-enriched diet alone improved spatial memory in a Y-Maze alternation test (p < 0.05) in mice. Additional physical enrichment enhanced the significance, although the overall benefit was virtually identical. Instead, physical enrichment improved motor performance in a Rotarod experiment (p* < 0.05) which was unaffected by walnuts alone. Bioactive oxylipins like hydroxy-polyunsaturated fatty acids (OH-PUFA) derived from linoleic acid (LA) were significantly increased in brain (p** < 0.01) and liver (p*** < 0.0001) compared to control mice, while OH-PUFA of α-linolenic acid (ALA) could only be detected in the brains of mice fed with walnuts. In the brain, walnuts combined with physical activity reduced arachidonic acid (ARA)-based oxylipin levels (*p* < 0.05). Effects of walnut lipids were not linked to mitochondrial function, as ATP production, mitochondrial membrane potential and mitochondrial respiration were unaffected. Furthermore, common markers for synaptic plasticity and neuronal growth, key genes in the regulation of cytoprotective response to oxidative stress and neuronal growth were unaffected. Taken together, walnuts change the oxylipin profile in liver and brain, which could have beneficial effects for healthy ageing, an effect that can be further enhanced with an active lifestyle. Further studies may focus on specific nutrient lipids that potentially provide preventive effects in the brain.

**Supplementary Information:**

The online version contains supplementary material available at 10.1007/s12017-020-08639-7.

## Introduction

**Fig. 1:**
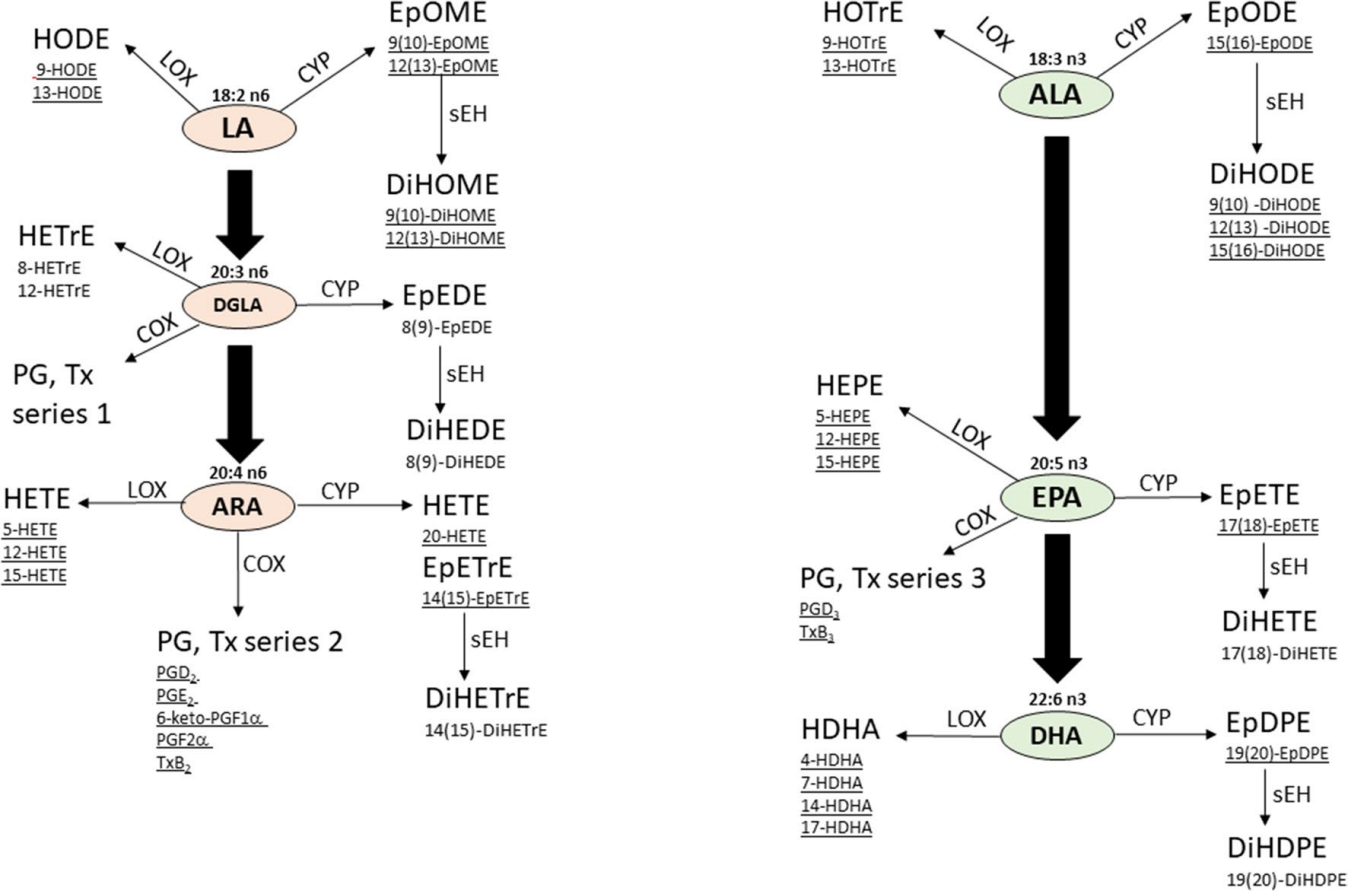
Simplified PUFA synthesis and origin of oxylipins analysed within this study formed by cyclooxygenase (COX), lipoxygenase (LOX) and cytochrome P450 monooxygenase (CYP) (Gabbs 2015; Ostermann et al. 2017b; Coras 2020). Bold arrows connecting PUFA indicate a multiple-step elongation process between each of them. Thin arrows indicate oxylipins formed by the exemplary indicated enzymes from the PUFA in single or multiple-step reactions. It should be noted that several of the oxylipins can also be formed by other pathways as well as autooxidation. n6-PUFA are shown on the left, n3-PUFA on the right. The oxylipins act as potent lipid mediators showing diverse biological activity ranging from pro-inflammatory (e.g. PG, LT) to analgesic, vasodilatory, anti-inflammatory action (Epoxy-PUFA). Oxylipins from n3-PUFA are commonly considered to be less potent inflammatory mediators compared to n6-PUFA oxylipins or even anti-inflammatory. Displayed are only a few selected oxylipins, not a complete set of all oxylipins known to be produced. Data for underlined oxylipins can be found in Table 5 or Fig. 5

In physiological ageing as well as in neurodegenerative diseases like Alzheimer’s disease (AD) or Parkinson’s disease, mitochondrial dysfunction (Swerdlow et al. 2014) and chronic inflammation (Minciullo et al. 2016) appear to play key roles in cognitive decline and decreased motor function. Hallmarks of mitochondrial dysfunction include changes of the oxidative phosphorylation system (OXPHOS) leading to reduced complex activity, depolarization of the mitochondrial matrix and inner membrane space and therefore to a reduced ATP production (Grimm et al. 2016). Changes to complex-I and -III of the OXPHOS system also lead to increased reactive oxygen species (ROS) production, potentially accelerating processes leading to neuronal loss. Low-grade chronic, systemic inflammation during ageing, often referred to as “inflammaging”, is based on the body’s decreasing ability to ameliorate inflammatory events, leading to an increased production of pro-inflammatory cytokines like IL1β or TNF1α or prostaglandins such as PGE_2_ or thromboxanes (Minciullo et al. 2016; Neves and Sousa-Victor 2019). Arachidonic acid (ARA) is predominantly metabolized by cyclooxygenases (COX), lipoxygenases (LOX), or cytochrome P450 monooxygenases (CYP) to a diverse pattern of eicosanoids with a wide range of biological roles (see Fig. 1). Several products, particularly prostaglandins as well as leukotrienes act pro-inflammatory (Nayeem 2018). The same enzymes also oxidize n3-fatty acids like α-linolenic acid (ALA), eicosapentaenoic acid (EPA) or docosahexaenoic acid (DHA). However, most of these metabolites are widely considered to exhibit anti-inflammatory effects (Nayeem 2018). Since both, n6- and n3-fatty acids (Kutzner et al. 2017) compete as substrates for COX, LOX and CYP, dietary supplementation with specific fatty acids is suspected to modify the metabolite profile in the body (Nayeem 2018; Ostermann et al. 2017a).

ALA is a precursor of long-chain n3-polyunsaturated fatty acids (n3-PUFA) like EPA and DHA and cannot be produced by the body itself. Supplementation of ALA has shown to increase brain DHA levels (Eckert et al. 2010) and anti-inflammatory oxylipins (Desai et al. 2016). Furthermore, ALA was also found to reduce Aβ induced neuroinflammation and cognitive dysfunction in C57BL/6N mice (Ali et al. 2020). ALA also attenuated its toxicity in SH-SY5Y cells (Litwiniuk et al. 2020). It was further found to increase brain plasticity in C57BL/6N mice following ALA injections (Blondeau et al. 2009) and reduce PD symptoms in *C. elegans* model (Shashikumar et al. 2015). In general, we and others reported that other n3-PUFA like DHA and EPA exhibit protective effects against neurodegenerative diseases like PD (Bousquet et al. 2008), but also support cognitive development in infants (Dyall 2015; Weiser et al. 2016) and be beneficial maintaining it during ageing (Cutuli 2017). Rey et al. also reported that feeding of mice with a n3-PUFA rich diet still retained their anti-inflammatory oxylipin profile following LPS-induced inflammation (Rey et al. 2019) compared to n3-PUFA deficient mice.

Walnuts (*juglans*) consist of about 47% PUFA (Ros 2009) and have one of the highest concentration of ALA of all edible nuts (Carey et al. 2013). Favourably, they also have a high ratio of n3-PUFA/n6-PUFA of 1:3 to 1:4 (Vinson and Cai 2012; Poulose et al. 2014). n3-PUFA are known to reduce the production of peptide amyloid-beta (Aβ) (Emendato et al. 2016; Thomas et al. 2015), commonly associated with AD, and additionally improve health of brain cells (Carey et al. 2013). For example, in accordance with Cole et al. we reported that DHA improved the fluidity of neuronal membranes (Cole et al. 2009; Eckert et al. 2011). Furthermore, n3-PUFA and their metabolites modulate gene expression in the brain including genes involved in mitochondrial biogenesis, ATP production and oxidative metabolism (Eckert et al. 2013).

Intake of walnuts was associated with improved working memory in elderly people (Valls-Pedret et al. 2012,2015). Besides PUFAs, walnuts are rich in polyphenols and vitamin E (McKay et al. 2010; Bourre 2006a, b ), which are also important for a healthy brain, as their antioxidative properties are an intrinsic counteraction to n3-PUFAs susceptibility to lipid peroxidation (Carey et al. 2013; Cole et al. 2009; Rosales-Martínez et al. 2017) or their effect on mitochondria affected survival in mice and nematodes (Dilberger et al. 2019).

Several studies have shown that a physically active lifestyle encouraged by an enriched environment attenuated cognitive decline in ageing and AD models (Maesako et al. 2012; Costa et al. 2007; Baraldi et al. 2013; Verret et al. 2013) and improves mitochondrial function in the brain (Schaffer et al. 2012; Steiner et al. 1985; Lores-Arnaiz et al. 2010; Asseburg et al. 2016). Moreover, acute and chronic exercise mobilized oxylipins that are most related to inflammatory processes, tissue repair or oxidative stress (Signini et al. 2020).

This study aimed to investigate whether a 6% walnut-enriched diet reflecting an uptake of 28 g/day for humans (Willis et al. 2009), could lead to a shift in the oxylipin profile and also affect cognition of mice by improving mitochondrial function and neuronal growth. The study design is based on our previous work investigating the effect of rice bran extracts in aged Naval Medical Research Institute (NMRI) mice (Hagl et al. 2016). Old and young control mice were fed with a control diet mainly based on oleic acid. Nuts and oleic acid have already been shown to positively enhance cognition in elderly subjects (Martínez-Lapiscina et al. 2013) and benefit other parameters like inflammation or blood pressure (Sales-Campos et al. 2013).

In rats, this diet has already shown to improve cognition i.e. by enhancing memory, but also improved animals’ motor function (Willis et al. 2009; Haider et al. 2011). In another study, Pandaresh et al. reported that a diet enriched with walnuts affected ROS production and markers of oxidative stress positively after 5–15 months of feeding in a murine model of AD (Pandareesh et al. 2018). Oxidative and nitrosative stress are a hallmark not only of AD, but also of the physiological ageing process (Dilberger et al. 2019; Jiménez-Jiménez et al. 2016). A study by Liu et al. found that oral gavage of 600 mg/(kg*d) of walnut kernels would protect mice in an ageing model using d-galactose-induced liver and brain damages (Liu et al. 2019), proposedly by enhanced ATP production and normalized acetylcholinesterase activity. Currently, data regarding synergistic effects of exercise and PUFAs, in form of walnuts, regarding oxylipin metabolism, cognitive function and mitochondrial performance are very limited. In this regard, this study might provide valuable insights and a foundation for further research in this area on the effect of walnuts, exercise and ageing.

## Methods

### Chemicals

All chemicals used for this research were of highest purity available and purchased from either Sigma Aldrich, Merck or VWR. Oxylipin standards and internal standards were acquired and prepared as previously described (Kutzner et al. 2019; Rund et al. 2018; Ostermann et al. 2020; Koch et al. 2020). Aqueous solutions were prepared with type-1 ultrapure water.

### Animals

Female NMRI (Naval Medical Research Institute) mice were acquired from Charles River (Sulzbach, Germany) and kept in the animal facility of the pharmacological institute of the Goethe University Frankfurt am Main until they reached the starting age of 12 months. Female mice were chosen because of later re-housing, which could have resulted in increased injury of male mice as they tend to show increased rivalry in a new environment. Initial Y-maze and Rotarod tests were conducted in order to divide all mice into 3 homogenous groups à 15 mice. After that, mice were moved into new cages in order for all groups to be of equal skill at the start of the trial. One group (oldCon) received a control diet (control diet C1000, Altromin, Lage, Germany) modified with sun flower oil (Lamotte OILS, Bremen, Germany) as basis for fats resulting in a total amount of 5.8% fat, for 6 months The composition of the diet including the fatty acid pattern are shown in Tables 1 and 2. Two groups (Wal and WalEE) received an identical pelleted diet enriched with 6% walnuts (Walnut, 60 g/kg diet) (provided by the California Walnut Commission, California, USA) for 6 months. Total fat amount of walnut diet was 5.8% and consisted of mainly oleic acid, n6-PUFAs LA and the n3-PUFA ALA. A detailed list of all fatty acids contained in the diets can be found in Table 2. Additionally, one walnut group (WalEE), were housed in bigger rat cages with an enriched environment in form of running wheels, houses and other objects encouraging physical activity. A young control group (youCon) of 15, 3-week-old mice was added 3 months after the start of the feeding period to ensure that feeding periods ended at the same time point. All mice had ad libitum access to diets and water and the young control group received the same diet as the aged control group. Behavioural testing was performed again at the end of the feeding period before mice were sacrificed via decapitation. Cerebellum, brain stem and olfactory bulb were removed from the brain before it was dissected on ice for further experimentations. Liver tissue was snap frozen after dissection.

**Table 1:** General components of C1000 diet. Modification of diets were based on fat composition (according to the manufacturer)

General Components	Control diet (sunflower oil) [%]	6% walnut-enriched diet [%]
Protein	17	18
Fat	5.7	5.8
Carbohydrates	47	45
Ash	5.5	5.9
Fibre	3.1	3.1
Sugar	11	11
Vitamin E [µg]	180	160

**Table 2:** Detailed fat composition of each diet. Fatty acid content was determined as methyl esters by gas chromatography-mass-spectrometry after lipid extraction and derivatization (see material & methods)

Fatty acid	Control diet (sunflower oil) [%]	6% walnut-enriched diet [%]
Palmitic acid	3.37	7.71
Stearic acid	1.12	1.85
Oleic acid	89.0	44.2
Vaccenic acid	1.62	1.42
Linoleic acid	4.90	39.5
Linolenic acid	–	5.35
Total fats	5.70	5.80

### Determination of Fat Composition and Quantification

Fatty acid composition and quantification was carried out following a protocol of Weibull-Stoldt’s according to AOAC 963.15 (Official Methods of Analysis of AOAC International. 1995). In brief, diet pellets were finely grounded in liquid nitrogen and hydrolysed in HCl for 30 min. Fatty acid extraction was performed using a Soxhlet Extraction System (Gerhardt Analytical Systems, Königswinter, Germany) in light petroleum.

Prior to analysis via GC/MS, fatty acids were methylated with BF_3_ (20% in MeOH). Before injection, GC/MS samples were further diluted 1:10 with isooctane. Only peaks > 0.05% of the highest peak found were considered for analysis. NIST Mass Spectral Library 2.0 g was used for identification of compounds.

### Open Field

For open field experiments, mice were placed in the middle of a 45 × 45 cm big arena. Mice were then allowed to freely roam around in the area for 5 min. Mice were filmed via a camera placed above the arena pointing downwards and recorded with TSE VideoMot 3D Classic V8.02. Later, videos were blinded by a second party, reformatted several times using DaVinci Resolve 15 (Blackmagic Design) and finally analysed using MouseMove Version 1, an open source program used to analyse the movement of mice (Samson et al. 2015).

Data for travel distance, speed, left and right turns stop fraction and number of faecal droppings were analysed using GraphPad Prism version 8.2. for Windows (GraphPad Software).

### Y-Maze

Cages were blinded by a second party, before the start of the experiment. Mice were placed in one arm of a Y-shaped maze. On the intersection of all three arms, visual indicators in form of shapes were placed in order for the mouse to orient itself. A mouse was placed into one randomly chosen arm of the maze and left to explore the maze for 5 min without further disturbance of the experimenter. Entry into a new arm, labelled A, B or C, was documented. Later the number of entries was determined as well as the order in which a mouse traversed the maze. A full alternation was defined as a mouse visiting all 3 (e.g. A → B → C) arms, before returning to an already visited one. If the mouse entered arm A next (A → B → C → A), a second alternation would be counted (A → B → C & B → C → A) Alternation rate is given as ((number of alternations/total number of possible alternations) * 100).

### Rotarod

The Rotarod (Accelerating Rota Rod, Panlab/Harvard Apparatus) was set to a base speed of 13 rpm. The speed increased over the course of one min to a maximum speed of 40 rpm. At base speed, mice were placed on the rod and the timer was started. The timer was stopped if a mouse fell from the rotating rod onto a pressure plate. If a mouse did not fall from the rod after 2 min, the test was also stopped.

Every mouse was trained 3 times for 2 consecutive days. Mice had a 15 min break between each training run. The final test was performed on the third day. The latency to fall was calculated as the mean time to fall from 3 runs on the final day of testing. Experimenter was blinded to which mice he was testing.

### Passive Avoidance

In order to investigate the mice’s ability to remember a negative event, a passive avoidance experiment was performed. The passive avoidance chamber (Passive Avoidance Step-trough New Model, Ugo basile) consists of two chambers. One chamber is illuminated by a bright light of 1,350 lm, the other chamber is dark. Both chambers are separated by a wall with a door. On the first day of the experiment, a mouse was placed in the bright chamber. Shortly after, the door to the dark chamber is opened and the time of how long it took the mouse to completely enter the dark chamber was recorded. Once it was inside, the door closed and a small electrical stimulus of 0.5 mA was applied to the mouse. The mouse was then removed from the apparatus and placed back into its cage. On the second day, the mouse was again placed into the chamber. This time, the door to the dark chamber was opened from the start. Upon entering the dark chamber there would be no electrical stimulus. The time it took for the mouse to enter the dark chamber was recorded. As a healthy mouse would remember the stimulus, it would generally stay in the bright chamber for a longer period of time.

### Preparation of Dissociated Brain Cells From Freshly Isolated Brains

Dissociated brain cells (DBC) were prepared from one brain hemisphere according to a previously published protocol (Franke et al. 2007). For MMP measurements, DBCs were diluted in un-supplemented DMEM (Gibco, Thermo Scientific) and seeded into 24-well plates (250 µL per well). For ATP measurements, the diluted DBCs were seeded in 96-well plates (50 µL per well). Additionally to basal MMP and ATP levels, a subset of DBCs was also treated with sodium nitroprusside (SNP) (2 mM for MMP and 0.1 mM for ATP) to simulate increased nitrosative stress found in ageing. DBCs were than incubated at 37 °C and 5% CO_2_ for 3 h before measurement.

### Mitochondrial Membrane Potential (MMP)

MMP was measured using fluorescence dye rhodamine 123 (R123). Dissociated brain cells of freshly dissected brain were incubated at 37 °C and 5% CO2 for 15 min with 0.4 µM R123. Cells were than centrifuged at 750 g for 5 min and washed with HBSS buffer (supplemented with Mg^2+^, Ca^2+^ and HEPES; pH 7.4; 37 °C). Cells were resuspended in fresh HBSS buffer before R123 fluorescence was determined. The excitation wavelength was set to 490 nm and the emission wavelength to 535 nm on a ClarioStar plate reader (BMG Labtech, Ortenberg, Germany). The fluorescence was measured 4 times and normalized to protein contents assessed via BCA method.

### ATP Levels

ATP concentrations were determined using an ATPlite Luminescence Assay System (Perkin Elmer, Rodgau-Jügesheim, Germany), which is based on the light emission of luciferin upon reaction with ATP. The 96-well plate was removed from the incubator and allowed to cool to room temperature for 10 min. Following incubation with lysis buffer for 10 min in the dark the monitoring reagent was added to the wells. The plate was again incubated in a dark environment at room temperature for 5 min. The emitted light was assessed with a ClarioStar plate reader (BMG Labtech, Ortenberg, Germany). The results were adjusted to protein content.

### Isolation of Brain Mitochondria and High-Resolution Respirometry

Frontal half of one brain hemisphere was homogenized in 2 mL MiR05 (mitochondrial respiration medium developed by Oroboros (Gnaiger 2020)) containing EGTA (0.5 mM), magnesium dichloride (3 mM), lactobionic acid (60 mM), taurine (20 mM), potassium dihydrogen phosphate (10 mM), HEPES (20 mM), sucrose (110 mM) and essentially fatty acid free bovine serum albumin (1 g/L). Sample was than centrifuged at 1.400 g for 7 min at 4 °C to remove cell debris. Supernatant was centrifuged at the same conditions for 3 min, than at 10,000 g for 5 min and 4 °C to yield a pellet of crude mitochondria. For further isolation, a protease inhibitor (PI, complete tablets, Roche, Mannheim, Germany) was added to the medium. The mitochondrial pellet was resolved in MiR05 + PI and centrifuged again at 1,400 g for 3 min at 4 °C. Supernatant was again centrifuged at 10,000 g for 5 min at 4 °C to yield the pure mitochondria which were than resolved in 900 µL MiR05 + PI of which 80 µL were injected into the Oxygraph for high-resolution respirometry.

Investigation of the respiratory system was performed following a protocol created by Gnaiger et al. (Gnaiger 2020). After injection of mitochondria into the Oxygraph 2 k chamber (Oroboros, Innsbruck, Austria), complex-I (CI) activity was determined by addition of 5 mM pyruvate and 2 mM malate (CI_L_). Addition of 2 mM ADP reflected physiological CI_P_ activity. Subsequent addition of 10 mM succinate activated complex-II, resulting in full physiological respiration CI + II_P_. Mitochondrial integrity was examined via addition of 10 µM cytochrome C. Uncoupled CI + II_E_ was determined via stepwise addition of carbonyl cyanide p-(trifluoromethoxy) phenylhydrazone (FCCP, up to 1–1.5 µM). Uncoupled CII_E_ was measured following the addition of complex-I inhibitor rotenone (0.5 µM). Leak respiration (CII_L_) was achieved by addition of oligomycin (2 µg/mL) and residual oxygen consumption, caused by enzymes which are not involved in the electron transfer chain, was determined via by addition of 2.5 µM antimycin A. Finally, CIV_E_ activity was determined by addition of 0.5 mM tetramethylphenylenediamine (TMPD), an artificial substrate of complex IV and 2 mM ascorbate to regenerate TMPD. All data points were corrected for residual oxygen consumption and CIV_E_ was additionally corrected for autooxidation rate, determined via addition of excess NaN_3_ after the final step of the experiment.

### Citrate Synthase Activity

A small sample of mitochondria from the respirometry measurements was frozen in LN_2_ and stored at − 80 °C until assessment of the citrate synthases activity. Samples were allowed to thaw while a reaction medium (0.1 mM DTNB, 0.5 mM oxaloacetate, 50 µM EDTA, 0.31 mM acetyl coenzyme A, 5 mM triethanolamine hydrochloride and 0.1 M Tris–HCl) was mixed and heated to 30 °C for 5 min. A volume of 10 µL of the mitochondria sample was added to the reaction medium and the citrate synthase activity was determined photospectrometrically at 412 nm. Measurements were performed in triplicate.

### Protein Content

A small sample of mitochondria from the respirometry measurements or DBCs were frozen in LN_2_ and stored at -80 °C until assessment of protein contents. Samples were thawed and protein contents were determined using Pierce BCA Protein Assay Kit (Thermo Fisher Scientific, Waltham, MA, USA) according to manufacturer’s instructions. Absorbance was measured using a ClarioStar plate reader (BMG Labtech, Ortenberg, Germany).

### Quantitative Real-Time PCR (qRT-PCR)

Freshly dissected brain tissue (~ 20 mg) from the rear part of a brain hemisphere was stabilized with RNAlater (Qiagen, Hilden, Germany), frozen in LN_2_ and stored at -80 °C until experimentation. Total RNA was isolated from brain tissue using the RNeasy Mini Kit (Qiagen, Hilden, Germany) according to the manufacturer’s instructions.

Assessment of purity and quantification was achieved using a NanoDrop 2000 × spectrophotometer (Thermo Fisher Scientific, Waltham, MA, USA). RNA was treated with TurboDNA free Kit (Qiagen, Hilden, Germany) to further improve quality of isolated RNA. cDNA was synthesized using an iScript cDNA synthesis kit (BioRad, Munich, Germany) according to manufacturer’s instructions. To perform qRT-PCR, SYBR Green technology was used on a CFX96 Touch real-time PCR detection system (BioRad, Munich, Germany). qRT-PCR was performed in a total volume of 10 µL assessing each sample in triplicate. Primer sequences, concentrations, product sizes and PCR conditions can be found in Table 3. Gene expression was analysed using the 2(-∆∆Cq) method with a BioRad CFX manager (BioRad, Munich, Germany) and normalized to phosphoglycerate kinase 1 (PGK1) and beta-2-microglobulin (B2M) expression levels. All used primers for qPCR were acquired from Biomol (Hamburg, Germany).

### Determination of Free Oxylipin Profile in Brain and Liver

Free oxylipins were analysed as previously described (Kutzner et al. 2019; Rund et al. 2018,2019). In brief, 10 µL of internal standards, butylated hydroxy toluene and enzyme inhibitors were added to ~ 20–50 mg tissue. The samples were homogenized in 300 µL methanol using a ball mill. Samples were centrifuged, the supernatants were collected and loaded onto the pre-conditioned solid phase extraction (SPE) cartridges. Oxylipins were extracted by SPE using a C8/anion exchange cartridge material (Bond Elut Certify II, Agilent, Waldbronn, Germany). The eluate was evaporated and reconstituted in methanol containing second internal standards. Samples were analysed by means of LC–MS/MS (QTRAP, Sciex, Darmstadt, Germany) with negative electrospray ionization. Detection was carried out in scheduled selected reaction monitoring mode.

### Data Handling and Statistics

Unless stated otherwise, data are presented as mean ± SEM. Statistical analysis was performed using either a student’s t-test or an one-way ANOVA followed by Dunnett’s post-test performed with GraphPad Prism version 8.2 for Windows (GraphPad Software).

Mice were excluded from the experiments if they did not appear to be of sufficient health for behavioural testing. Outliers were removed according to ROUT’s outlier test applying Q = 1%. For oxylipin analysis, statistical evaluation was done if > 50% of samples met the lower limit of quantification (> LLOQ) and if the mean of all samples was still > LLOQ. All samples which did not reach the LLOQ were set to their specific limit of detection (LOD) for calculation. If the concentration of an oxylipin was < LLOQ for > 50% of the group’s mice, the analysed group was excluded from analysis and “ < LLOQ” is displayed instead.

## Results

### Treatment

After reaching adulthood the mice’s bodyweights stayed stable for the whole feeding period. Mice in each group showed a similar range of bodyweights, although they differed by around 15–20 g inside of each group. Weight changes for mean bodyweights and individual bodyweights can be found in supplementary Table S10. Mice from both Wal and WalEE groups were around 1.5–2 g heavier than the oldCon mice during the whole feeding period.

### Behavioural Tests

**Fig. 2:**
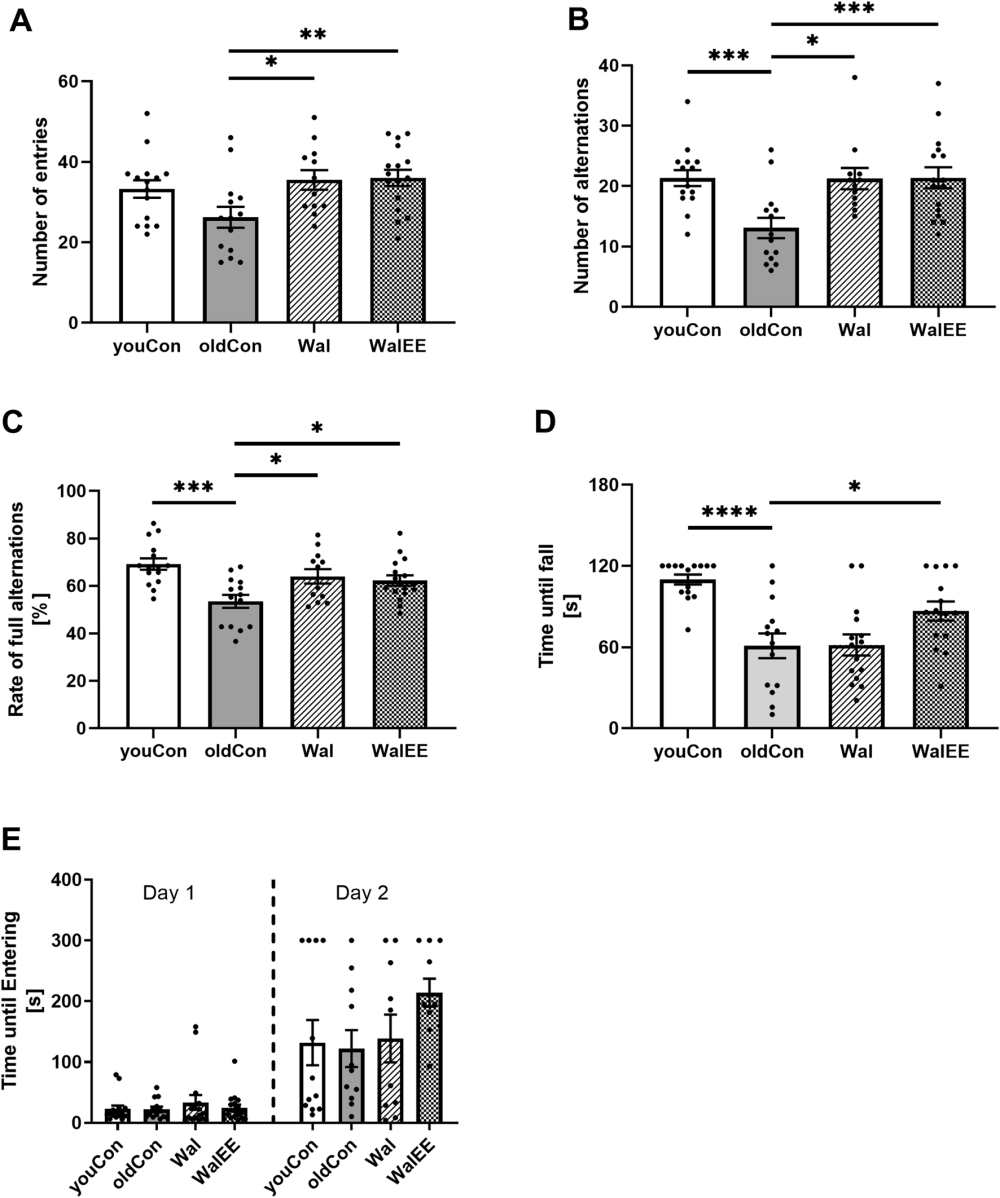
Y-Maze alternation test. **a** Number of entries into the three different arms of the Y-Maze. Duration of Y-Maze experiment was 5 min. **b** Number of full alternations. One full alternation is defined as a mouse entering all arms of the maze before re-entering the already visited one. **c** Alternation rate of Y-Maze test, calculated as the number of full alternations divided by the number of entries. **d** Rotarod test. Time in [s] of mice being able to stay on the rotating rod. Speed of the rod gradually increased until 60 s into the experiment; experiment was stopped if mice were able to stay on the rod for 120 s. **e** Passive Avoidance test. Mice were put in an illuminated chamber and time was measured until they retreated into a dark chamber. On day one, a weak stimulus was applied upon entering the dark chamber. On the second day, time until mice entered the dark chamber was recorded to investigate mice’s memory. Displayed are mean ± SEM of *n* = 12–15 in **a–d** and *n* = 10–12 in **e**. Full range of data is displayed using dots for each individual mice. Statistical significance was tested via one-way ANOVA and post hoc Dunnett’s test comparing all groups with oldCon (*p < 0.5, **p < 0.01, ***p < 0.001, ****p < 0.0001). oldCon = aged Control; youCon = Young control; Wal = Walnut group; WalEE = Walnut + Enriched Environment group. Parameters for the statistical tests can be found in the supplementary Table S1

Aged mice showed significant deficits in Y-Maze alternation (p*** = 0.0009) and Rotarod tests (p**** < 0.0001) compared to young mice (Fig. 2a–d). A 6% walnut-enriched diet showed significantly higher numbers of entries in Y-Maze test (p* = 0.0237), but did not have an effect on motor function in Rotarod test (*p* = 0.9999). Number of alternations (Wal: p* = 0.0157; WalEE: p*** = 0.0007) and alternation rate (Wal: p* = 0.0207; WalEE: p* = 0.0421) were significantly increased in both Wal and WalEE. Although the intervention group with additional physical enrichment showed the same results, they were more homogenous among all mice leading to a higher significance in the number of alternations (p*** = 0.0007) but not for the alternation rate (p* = 0.0421). While motor functions in Wal mice were virtually identical to aged control mice, physical enrichment attenuated the age-dependant decrease in motor function significantly (p* = 0.0381).

Generally all groups in the Passive Avoidance test (Fig. 2e) showed a higher delay before entering the dark chamber on the second day suggesting that mice remembered the adverse event. However, only trends could be identified by statistical evaluation. Walnut intervention alone did not appear to affect passive avoidance performance (*p* = 0.9716), but physically active mice tended to stay in the illuminated chamber longer than aged mice (*p* = 0.1507). Young control mice only showed virtually identical behaviour like aged mice (*p* = 0.9930).

We also performed an open field experiment to examine the general behaviour and activity of mice. We assessed mice’s travelled distance, stop fraction, average speed and turn. We found only little numerical differences in all measured parameters. Aged control mice showed a small trend to underperform, while other groups were virtually identical. Quantity of faecal droppings during the Open Field experiment is often linked to the emotionality of the animals (Seibenhener and Wooten 2015). Our results suggest that the interventions did not affect mice’s sensitivity to emotional/stressful events in a measurable way. Results for Open Field experiments and faecal droppings can be found in supplementary Fig. S2 and supplementary Table S1 and supplementary Table S3.

### Mitochondrial Parameters

**Fig. 3:**
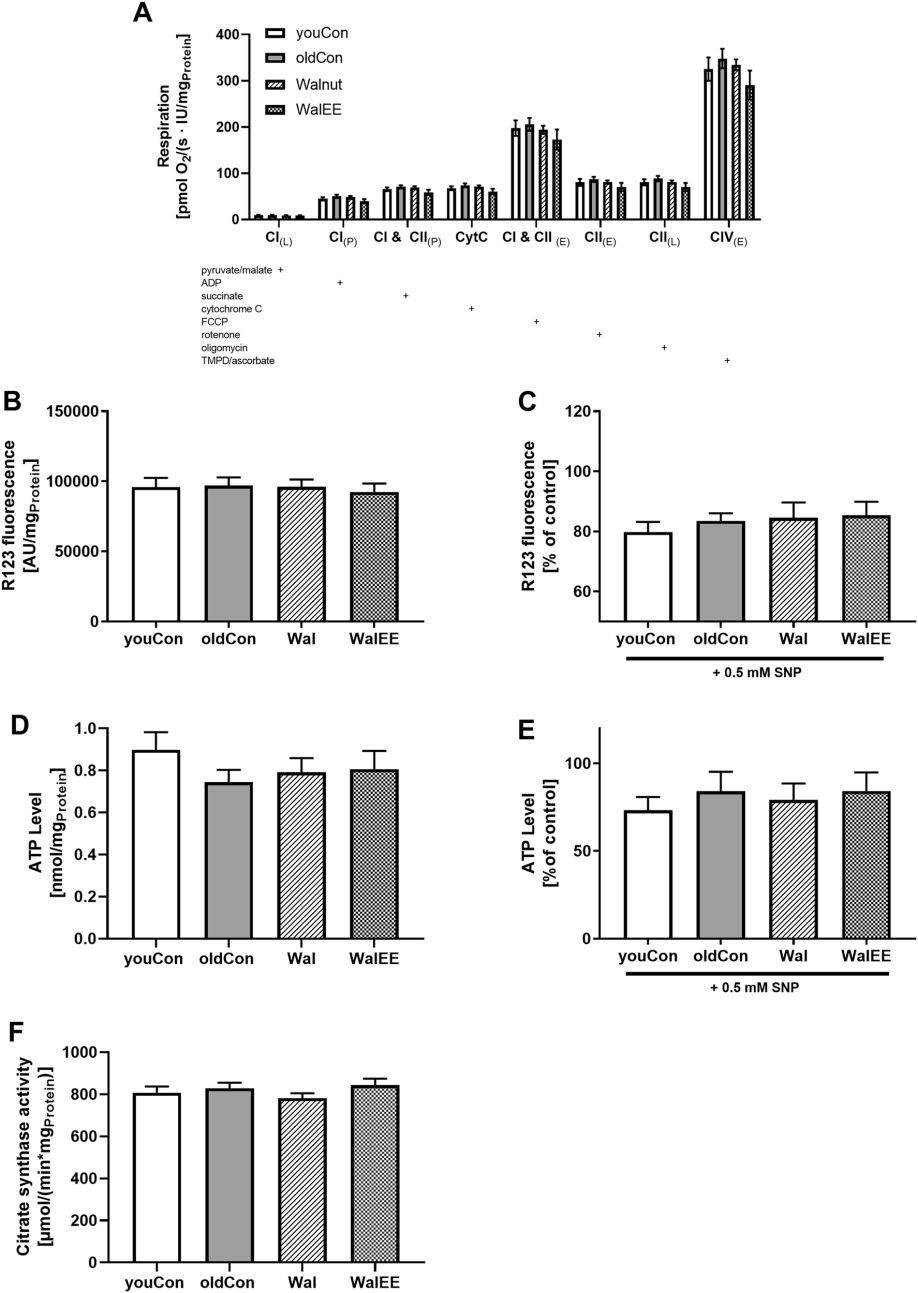
**a** Oxygen consumption of mitochondria isolated from the brain adjusted to protein. Activity of OXPHOS complexes were assessed via addition of several substrates, inhibitors or uncouplers. Which substance was added in which stage of the experiment is marked with “ + ”; *n* = 12. **b** Basal MMP levels were measured as R123 fluorescence after incubation of DBC samples. **c** MMP levels after 0.5 mM SNP-induced nitrosative stress. **d** Basal ATP concentration measured in µM per mg protein in the DBC samples. Measured signal stems from bioluminescence reaction of luciferin and ATP. **e** ATP levels after SNP-induced nitrosative stress. DBCs have been incubated for 3 h prior to measurement. **f** Citrate synthase activity as an indicator of mitochondrial content. Amount of protein was measured via BCA method. Displayed are means ± SEM. Statistical significance was tested via one-way ANOVA with Dunnett’s post hoc test using oldCon as reference for statistical comparisons. Each group in C-F consisted of 12–15 female NMRI mice. Parameters for the statistical tests can be found in the supplementary Table S2

Complex activities of the oxidative phosphorylation system of isolated mitochondria from freshly dissected mice brain showed no differences between the groups, indicating that the interventions did not have an effect on mitochondrial respiration (Fig. 3a). Only a trend for WalEE mice to have reduced complex activity could be found. As seen in Fig. 3f, using citrate synthases activity as a marker for mitochondrial mass, mitochondrial content also seemed to be unaffected by the interventions.

Furthermore, MMP (Fig. 3b), which was determined as R123 fluorescence, was unaffected by the interventions, supporting the next to identical complex activity of the OXPHOS. Sodium nitroprusside (SNP) was used to induce nitrosative stress to the DBCs as the generation of reactive nitrogen species (RNS) is a common process in ageing. After addition of 0.5 mM SNP (Fig. 3c) and incubation for 3 h, a decrease in MMP of approximately 15% was observed. Neither interventions, however, lead to an improved reaction to SNP-insult.

Although ATP is mainly produced in the OXPHOS, a minor part of ATP is also generated, for example, during glycolysis. Since respiration and MMP were unaffected, we measured ATP levels. As displayed in Fig. 3d, ATP levels were not increased in either intervention group. After insult with SNP (Fig. 3e), ATP levels of all groups were reduced by around 40% to 33% compared to untreated DBCs, whereas walnut interventions or additional physical enrichment had only little to no effect on the induced damages (Table 3)

**Table 3:** Primer sequences, manufacturer’s, product sizes (bp), concentrations (conc.) and programme used for qRT-PCR measurement

Primer	Sequence	Size [bp]	conc [µM]	Annealing Temp (time) ( no. of cycles)
PGK1	5′-GCAGATTGTTTGGAATGGTC-3’ 5′-TGCTCACATGGCTGACTTTA-3’	185	0.4	58 °C (45 s), (45x)
B2M	5′-GGCCTGTATGCTATCCAGAA-3’ 5′-GAAAGACCAGTCCTTGCGA-3’	198	0.4	58 °C (45 s), (45x)
BDNF	5′-GATGCCAGTTGCTTTGTCTT-3’ 5′-ATGTGAGAAGTTCGGCTTTG-3’	137	0.1	58 °C (45 s), (42x)
NGF	5′-ATCAAGGGCAAGGAGGTGACAG- ‘3 5′-GAGTTCCAGTGTTTGGAGTCGATG-3 ‘	143	0.15	62 °C (20 s), (40x)
NRF2	5′-GATCTCCTCGCTGGAAAAAG-3’ 5′-GTCACTGGGCTCTGCTATGA-3’	187	0.4	56 °C (30 s), (50x)
CREB1	5′-TAGCTGTGACTTGGCATTCA-3’ 5′-TTGTTCTGTTTGGGACCTGT-3’	184	0.5	58 °C (45 s), (45x)
Synaptophysin	5′-TTTGTGGTTGTTGAGTTCCT-3’ 5′-GCATTTCCTCCCCAAAGTAT-3’	204	0.1	58 °C (45 s), (42x)
Gap43	5′-AGGGAGATGGCTCTGCTACT-3’ 5′-GAGGACGGGGAGTTATCAGT-3’	190	0.15	58 °C (45 s), (42x)
Keap1	5′-ATGGCCACACTTTTCTGGAC-3’ 5′-TCCTGTTGTCAGTGCTCAGG-3’	131	0.2	60 °C (45 s), (45x)
IL1β	5′-CCCAACTGGTACATCAGCAC-3’ 5′-TCTGCTCATTCACGAAAAGG-3’	180	0.3	58 °C (30 s), (45x)

### Gene Expression in the Brain

To gain further mechanistic insight we investigated the gene expression of several markers for neuronal growth, synaptic plasticity and antioxidant system (Table 4). CREB1, which is closely related to long time memory, was significantly decreased in aged mice compared to young mice (p* = 0.0149), WalEE was virtually identical with oldCon (*p* = 0.8025). In general, gene expression in aged mice showed trends to be decreased for all investigated genes, indicating possible reduced synaptic plasticity (Synaptophysin, GAP43), neuronal growth (NGF, BDNF) or response to oxidative stress (KEAP1, NRF2). Neither walnut-only nor WalEE interventions were able to attenuate this age-dependant reduction.

**Table 4:** Relative normalized mRNA expression of relevant genes in brain tissue. Data are adjusted to aged control group = 100%; *n* = 10; Displayed are means ± SEM; one-way ANOVA with Dunnett´s multiple comparison post hoc test using oldCon as reference for statistical comparison; normalized to the mRNA expression levels of beta-2-microglobulin (B2M) and phosphoglycerate kinase 1 (PGK1)

Gene	OldCon [%]	YouCon [%]	Wal [%]	WalEE [%]
Synaptophysin	100 ± 7.6	124 ± 8.0	99.3 ± 8.6	110 ± 6.4
NGF	100 ± 9.4	145 ± 17	97.9 ± 18	115 ± 13
BDNF	100 ± 27	228 ± 53	98.3 ± 28	115 ± 28
GAP43	100 ± 28	111 ± 18	86.6 ± 12	81.7 ± 13
KEAP1	100 ± 11	144 ± 21	112 ± 18	124 ± 14
NRF2	100 ± 13	152 ± 25	89.5 ± 14	120 ± 23
CREB1	100 ± 8.7	144 ± 9.1 * p = 0.0149	78.5 ± 15	111 ± 6.7
IL1β	100 ± 17	116 ± 20	101 ± 20	158 ± 36

Significance is displayed as: *p < 0.05 (oldCon vs youCon). Parameters for the statistical tests can be found in the supplementary Table S8

### Oxylipin Profile and Gene Expression in the Liver

As our data suggest that neither gene expression in the brain nor mitochondrial function were affected by the intervention diet, we looked at whether the diet was affecting fatty acid metabolism. For this reason, and to investigate whether the diets might have an effect on inflammatory processes, commonly increasing during the process of ageing, we explored the free, i.e. nonesterified oxylipin profiles in brain and liver tissue.

**Fig. 4:**
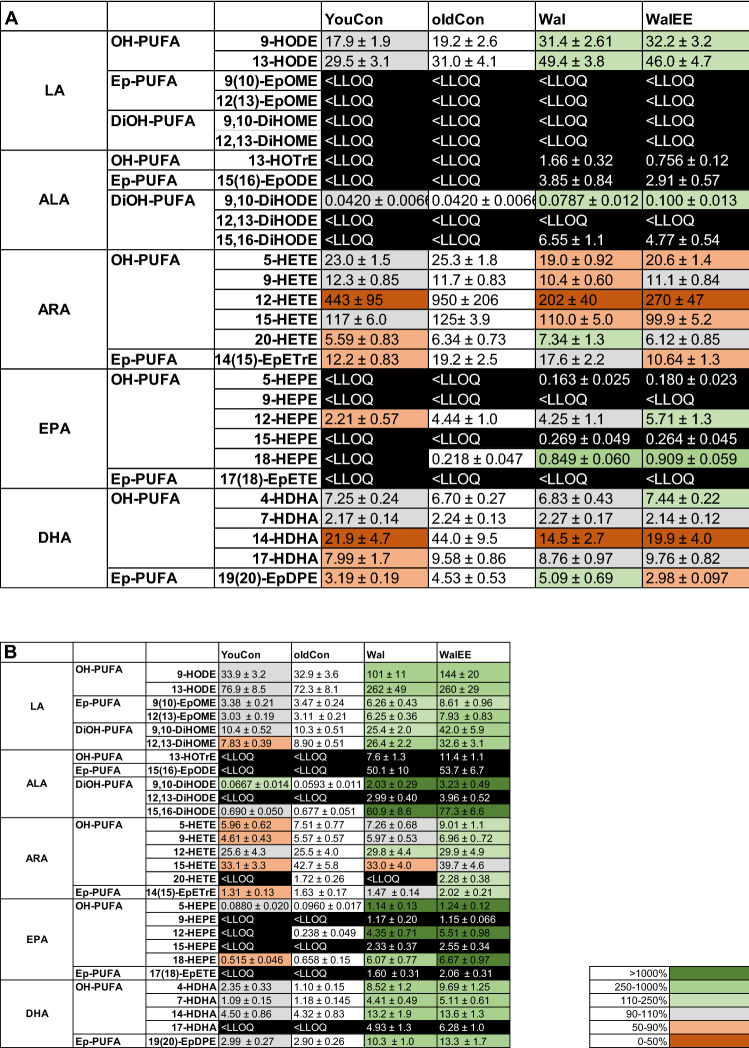
Heatmap of hydroxy-PUFA, epoxy-PUFA and dihydroxy-PUFA oxylipins derived from LA, ALA, ARA, EPA and DHA in brain (**a**) and liver (**b**). *Note* Data are displayed relative to results for old control group (oldCon, marked in white). Green colouring and colour strength indicates an increase compared to the aged control group, while brown colouring indicates a decrease. < LLOQ indicate oxylipin concentrations below the lower limit of quantification. Full disclosure of results including significance levels can be found in the supportive information/appendix

**Fig. 5:**
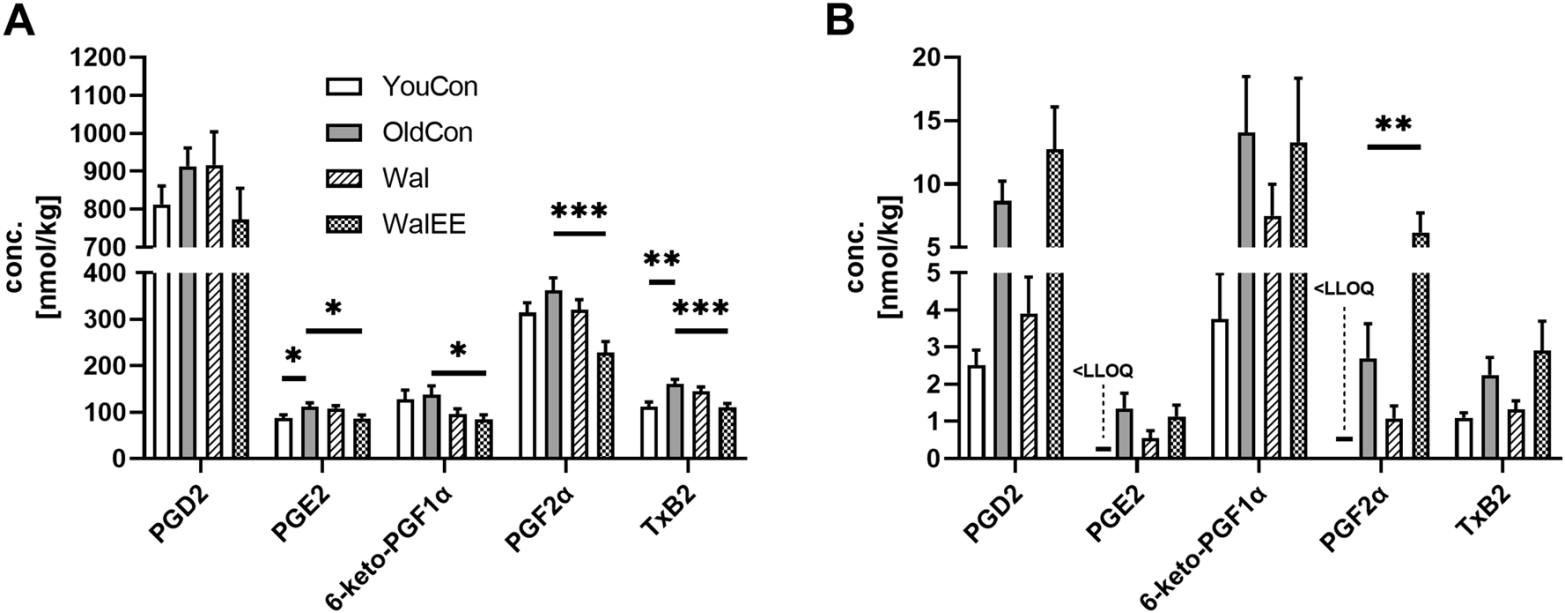
Concentration of ARA-derived prostaglandins in brain **a** and liver **b**; *n* = 15; Displayed are means ± SEM; the LLOQ is indicated if an analyte was < LLOQ in > 50% of the samples. One-way ANOVA with Dunnett’s multiple comparison post-test, comparing all groups with oldCon, was used to calculate significance; Significance is displayed as: *p < 0.05, **p < 0.01, ***p < 0.001. Statistical parameters can be found in supplementary Table S7

As seen in Table 5 and Fig. 4 as well as in Fig. 5, the diet’s effect varied between the two organs. Table 5 shows that in the liver hydroxy-PUFA (OH-PUFA) of walnut-related fatty acids were significantly increased compared to the aged control group (p*** < 0.001 to p**** < 0.0001). The same was observed for epoxy-PUFA (Ep-PUFA), as both intervention groups showed concentrations of 50.5 ± 10 nmol/kg (Wal) and 54.6 ± 6.7 nmol/kg (WalEE), while the concentration in oldCon was < LLOQ for all measured EpODEs. Similar results were found for LA (p* < 0.05 to p** < 0.01) and ALA (control < LLOQ)-based oxylipins in the brain. Interestingly, in the brain both intervention’s EPA and DHA OH-PUFA and Ep-PUFA were virtually identical compared to both control groups. Concerning the liver’s Ep-PUFA it is to note that for ALA, mainly 15(16)-EpODE could be detected in the intervention groups. The same could be said for their hydrolysis products, the DiHODEs, which can be formed by soluble epoxide hydrolase (sEH). Ep-PUFAs derived from EPA could only be found in intervention groups, but not in the control groups. The same is true for Ep-PUFA of DHA with the exception of 10(11)-EpDPE, 13(14)-EpDPE and 19(20)-EpDPE, which could also be found in the control group. In the brain, Ep-PUFAs of LA and EPA were < LLOQ in all groups and Ep-PUFAs of ALA, specifically 15(16)-EpODE, could only be detected in the intervention groups.

**Table 5:** Comparison of hydroxy- and epoxy-PUFA concentrations in liver and brain and between all different intervention groups. A: Sum of all brain hydroxy- and epoxy-PUFAs based on LA, ALA, ARA, EPA and DHA. B: Sum of all liver hydroxy- and epoxy-PUFAs based on LA, ALA, ARA, EPA and DHA; *N* = 15; Displayed are means ± SEM of each groups. Data were statistically compared via an one-way ANOVA with Dunnett’s post hoc test and oldCon group as reference (ref.)

A Brain	Hydroxy-PUFA [nmol/kg] derived from					Epoxy-PUFA [nmol/kg] derived from				
	LA	ALA	ARA	EPA	DHA	LA	ALA	ARA	EPA	DHA
oldCon ref	50.3 ± 6.7	< LLOQ	1480 ± 80 ref	4.95 ± 1.0	145 ± 16	< LLOQ	< LLOQ	40.6 ± 5.5 ref	< LLOQ	12.3 ± 1.7
YouCon	47.4 ± 5.0 n.s	< LLOQ	995 ± 110 *	2.89 ± 0.7 n.s	132 ± 17 n.s	< LLOQ	< LLOQ	25.3 ± 1.8 n.s	< LLOQ	8.29 ± 0.7 n.s
Wal	80.8 ± 6.4 **	1.56 ± 0.3	869 ± 78 **	5.85 ± 1.2 n.s	111 ± 14 n.s	< LLOQ	2.99 ± 0.5	37.7 ± 4.8 n.s	< LLOQ	14.4 ± 2.2 n.s
WalEE	75.8 ± 6.1 *	1.59 ± 0.3	778 ± 54 ***	7.44 ± 1.4 n.s	117 ± 10 n.s	< LLOQ	2.91 ± 0.6	22.1 ± 2.9 *	< LLOQ	7.74 ± 0.8 n.s

B Liver	Hydroxy-PUFA [nmol/kg] derived from					Epoxy-PUFA [nmol/kg] derived from				
	LA	ALA	ARA	EPA	DHA	LA	ALA	ARA	EPA	DHA
oldCon ref	114 ± 12	0.65 ± 0.2	150 ± 20 ref	4.47 ± 0.5	28.4 ± 3.9	6.57 ± 0.4	< LLOQ	3.95 ± 0.4 ref	< LLOQ	3.37 ± 0.3
youCon	120 ± 12 n.s	0.45 ± 0.1 n.s	110 ± 11 n.s	4.35 ± 0.6 n.s	26.1 ± 3.3 n.s	6.40 ± 0.4 n.s	< LLOQ	3.37 ± 0.4 n.s	< LLOQ	3.13 ± 0.3 n.s
Wal	347 ± 47 ****	15.1 ± 2.4 ****	128 ± 13 n.s	27.4 ± 2.9 ****	86.0 ± 9.5 ****	12.5 ± 0.8 ***	50.5 ± 10	3.28 ± 0.7 n.s	2.15 ± 0.3	13.3 ± 1.3 ****
WalEE	427 ± 50 ****	17.9 ± 1.5 ****	157 ± 17 n.s	33.5 ± 3.9 ****	97 ± 8.9 ****	16.5 ± 1.8 ****	54.6 ± 6.7	4.74 ± 0.6 n.s	2.45 ± 0.3	17.3 ± 2.0 ****

Significance between groups and the aged control group are displayed as *p < 0.05, **p < 0.01, ***p < 0.001, ****p < 0.0001. LLOQ = lower limit of quantification. Statistical parameters can be found in supplementary Table S4

As seen in Table 5, in the brain, OH-PUFA of ARA were significantly reduced in the groups that were fed the walnut-only diet (p** < 0.01). This change appears to originate mainly from the substantial reduction of 12-HETE. This effect was further enhanced in physically active mice (p*** < 0.001). The same trends can be found for brain Ep-PUFAs, but neither for OH-PUFAs or EP-PUFAs in the liver.

In general, across all groups, ARA-based prostanoid levels are around 80-fold higher in the brain tissue compared to the liver. As seen in Fig. 5, in the liver, the Wal group showed a general trend for reduced ARA-based pro-inflammatory prostanoids to around the level of the young control group (if detectable). In the brain, the oldCon group showed a trend for higher prostanoid concentrations compared to the youCon mice. While Wal mice did not change prostanoid levels, the WalEE group showed generally lower concentrations akin to those of the young mice. Still, these changes were only a trend in the case of PGD_2_, but significant for PGE_2_ (p* < 0.05), 6-ketoPGF1α (p* < 0.05), PGF2α (p*** < 0.001) and TxB_2_ (p*** < 0.001) compared to oldCon. Other prostanoids were virtually identical between each group. The effect of physical enrichment also varied between the organs. In the liver, WalEE showed similar prostanoid levels compared to old control, with the exception being PGF2α, which was increased compared to old control (p** < 0.01). Prostanoid levels in the Wal group, however, tended to be more in line with youCon.

Displayed in Fig. 4 is a heatmap showing increases or decreases of several OH- and Ep-PUFAs derived from LA, ALA, ARA, EPA and DHA relative to results of the oldCon group. Statistical evaluation of the analytes can be found in supplementary Table S5 for brain and supplementary Table S6 for the liver. Results for the liver show that in a similar vein as for the ARA-based prostanoids, an enriched environment in the WalEE group led to an all-encompassing increase of oxylipins compared to oldCon. The Wal group on the other hand showed results more akin to the youCon group, whose ARA-derived oxylipins were reduced compared to oldCon. Looking at data from brain tissue (see Table 5), we found that both intervention groups had reduced ARA-based oxylipins, while at the same time levels of oxylipins derived from LA were increased. Due to high biological variability of the outbred NMRI mice, this cannot be ambiguously said for the liver.

All changes considered, when looking at the n6/n3-PUFA-oxylipin ratio in Table 6, it is interesting to note that in the brain only trends for a changed ratio could be found, while the ratio in the liver samples was significantly different (p**** < 0.0001). Part of the reason for this is the high ARA-derived oxylipin concentration in the brain, which overshadowed smaller changes in ALA, EPA or DHA oxylipins.

**Table 6:** Ratio of n6/n3-PUFA oxylipins in brain and liver tissue. Displayed are means ± SEM of each groups; n = 14–15. Data were statistically compared via an one-way ANOVA with Tukey’s post hoc test and using aged control group (oldCon) as reference (ref.)

	oldCon	youCon	Wal	WalEE
Brain	22.6 ± 1.8 ref	19.7 ± 1.6 ns	20.4 ± 2.8 ns	16.3 ± 1.9 ns
Liver	6.29 ± 0.42 ref	5.31 ± 0.33 ns	1.47 ± 0.13 ****	1.79 ± 0.22 ****

Significance is displayed as: ****p < 0.0001

*oldCon* old Control, *youCon* Young control, *Wal* Walnut group, *WalEE* Walnut + Enriched Environment group

Statistical parameters can be found in supplementary Table S3

Looking at the expression of genes related to antioxidative capacity in the liver (Table 7), we see similar results as in the brain (Table 4). It is to note that Wal mice showed a significantly higher expression of KEAP1 gene, which is part of the KEAP1-NRF2 pathway of handling oxidative stress. NRF2, however, did not show an increase, but tended to be decreased in both interventions groups. However, the expression of Interleukin 1β as a marker for inflammation showed a greater variability in aged control mice resulting in a trend for a higher expression in the liver, which could potentially be reduced by the interventions. In the brain, the overall expression appears to be similar across all groups.

**Table 7:** Relative normalized mRNA expression of relevant genes from liver tissue. Data are adjusted to aged control group = 100%; *n* = 12; Displayed are means ± SEM; one-way ANOVA with Dunnett´s multiple comparison post-test comparing all groups with oldCon

Gene	oldCon [%]	youCon [%]	Wal [%]	WalEE [%]
KEAP1	85.3 ± 8.3	118 ± 16	158 ± 25*	138 ± 22
NRF2	100 ± 8.7	107 ± 11	77.0 ± 4.6	74.1 ± 5.4
CREB1	100 ± 14	133 ± 24	130 ± 21	97.5 ± 11
IL1β	100 ± 29	52.1 ± 12	56.2 ± 12	47.7 ± 7.0

Significance is displayed as *p* < 0.05 *; normalized to the mRNA expression levels of beta-2-microglobulin (B2M) and phosphoglycerate kinase 1 (PGK1)

*oldCon* old Control, *youCon *Young control, *Wal *Walnut group, *WalEE* Walnut + Enriched Environment group

Parameters for the statistical tests can be found in the supplementary Table S8

## Discussion

### Diet and Body Weight

A 6% walnut supplementation, which corresponds to 28 g/d for humans (Willis et al. 2009), has previously enhanced cognitive and motor function in humans and rodents (Willis et al. 2009; Haider et al. 2011). Mice adhered to the diets during the whole feeding period and gained only a little weight at the beginning. Starting at around week 3, mice kept their weight. As the NMRI mice were already 12 months old at the start of the feeding trial, weight differences and differences in their abilities were expected and adjusted for. Muthaiyah et al. who fed a 6% walnut-enriched diet to tg-AD mice also showed no to little differences in the body weight during the first 4–9 months of their diet (Muthaiyah et al. 2014). We therefore propose that the diet was generally well received and adhered to. For cognitive and motor testing, as well as metabolic changes or mitochondrial function, estrogenic state of female mice might affect the variability of data. As behavioural tests and sacrifice was conducted over a longer period of time, every state of the estrogenic cycle should be represented in the dataset. Furthermore, recent studies analysing data from multiple studies revealed that differences in variability between male and female mice are negligible (Prendergast et al. 2014; Smarr et al. 2019).

### Cognitive and Motor Function

In the Y-Maze alternation test, oldCon mice were significantly less active indicated by the number of entries in each arm. They also showed a reduced number of full alternations, reduced spatial memory compared to youCon mice. Spatial memory was significantly improved in the Wal and WalEE groups. Wang et al. also found that rats administered with 1.1 g/kg, 2.2 g/kg and 11 g/kg walnut oil showed improved cognitive performance in Morris Water Maze (MWM) experiments vaguely based on the concentration used (Wang et al. 2018). Other studies in rats and mice also found significant improvements using a 6% walnut-enriched diet in MWM test (Willis et al. 2009; Muthaiyah et al. 2014) and T-Water maze test (Muthaiyah et al. 2014). Multiple reports have shown that walnuts components, i.e. n3-FA (Carey et al. 2013; Eckert et al. 2013), vitamin E (Ashley et al. 2019) or other antioxidative compounds (Ren et al. 2018) like ellagic acid which is metabolized to urolithin A (Hayes et al. 2016; Gong et al. 2019), showed significant neuroprotective effects. Although here, walnuts alone did not show a significant impact on mice’s motor function, other studies have reported motoric improvements (Willis et al. 2009; Muthaiyah et al. 2014). Increased physical activity did not improve upon the effects seen in the Wal group, but significantly increased the motor functions in a Rotarod test compared to oldCon. Motor function of Wal mice, however, was unaffected. Physical activity has already been well established as beneficial to motor and cognitive function (Kim et al. 2019; Hatchard et al. 2014; Intlekofer and Cotman 2013), but to the best of our knowledge a combination of the two has not yet been reported. Open field experiments did not show any differences between the groups. Nevertheless these results do not indicate an effect of the interventions on mice’s level of curiosity or anxiety/emotionality (Seibenhener and Wooten 2015). The mean number of faecal boli of each of the groups reflects this finding. Muthaiyah et al., however, reported that mice fed with a 6% walnut-enriched diet showed significantly reduced response to emotional adverse events in an elevated-plus-maze experiment (Muthaiyah et al. 2014). Looking at our results for the Passive Avoidance test, which applies a small electrical stimulus to mice once they cross from a brightly illuminated chamber into a dark one, we could clearly see that all groups remembered the adverse event on the second day. Unexpectedly, however, in contrast to an earlier work (Hagl et al. 2016), no significant difference between youCon and oldCon were observed. As the behaviour of mice differed greatly in each group we propose that the high frequency of tests in a relatively short time (4 different experiments in 4 weeks) might have affected the mice’s behaviour.

### Mitochondrial Function in Mice

Since mitochondrial function plays a key role in neuronal processes during ageing and also the onset of neurodegenerative diseases (Grimm et al. 2016; Swerdlow et al. 2014), mitochondrial involvement was of key interest.

Multiple studies have shown that fat, the main component of walnuts, and especially n3-FA have a beneficial effect on mitochondrial parameters. Afshordel et al. reported that EPA and DHA from fish oil positively affected the OXPHOS activity in the brain of aged NMRI mice (Afshordel et al. 2015). Other groups also reported an effect of these fatty acids on mitochondrial membrane parameters and respiration in human skeletal muscle (Herbst et al. 2014). Rossignoli et al. also supplemented the diet of mice EPA, DHA or conjugated linoleic acid and found beneficial properties on mitochondrial energetics (Rossignoli et al. 2018). Across all mitochondrial parameters investigated in this study, however, we found no significant changes and at best only trends for an altered mitochondrial function in aged NMRI mice. Pandaresh et al. who fed tg-AD mice with 6% and a 9% walnut-enriched diet found that only the higher-dosed diet affected ROS levels and other markers for oxidative stress after 5 months of feeding, while the lower dosage which was similar to ours, showed its effect only after 10 and 15 months (Pandareesh et al. 2018). As mice in this study were not transfected and aged normally, mitochondrial dysfunction might not be as pronounced as in a dedicated AD mouse models. Combined with a feeding time of only 6 months this might also explain the lack of significant effects.

As ALA is also a major component of walnuts and precursor to both, EPA and DHA, we were interested to see, whether walnuts might attenuated NO-related stress in DBCs of aged mice. Nitric oxide radicals induce nitrosative stress (Carey et al. 2013) and are a crucial part during ageing and in the development of neurodegenerative diseases (Jiménez-Jiménez et al. 2016). In BV2 microglia cells both EPA and DHA have been found to attenuate NO-related damages (Lu et al. 2010; Moon et al. 2007). Introduction of nitrosative stress via sodium nitroprusside (SNP) showed that neither Wal nor WalEE mice protected MMP or ATP level. As the experimental design did not use a specific part of the brain, but a homogenate of brain cells due to the amounts necessary for experimentation, we propose that any effects could have been masked by unaffected areas of the brain.

Since the improvements seen in behavioural testing cannot be explained by an altered mitochondrial function, the expression of several genes related to synaptic plasticity, neuronal growth and antioxidant capacity were investigated. However, the overall effect on gene expression was limited. In this study, data suggest that youCon mice tend to show increased levels of neuronal growth factor, synaptophysin and BDNF as a marker for neuronal growth and synaptic plasticity. Since young mice are still in development, this difference was to be expected (Reutzel et al. 2020). Concerning the other assessed markers, no distinct effect of Wal or WalEE could be observed. Part of the reason for this might also be the high individual variability of the mice. As these results do not indicate altered cognitive functions, further behavioural testing should examine and verify the effects observed in the Y-Maze alternation test.

### Oxylipin Profiles

Next, we investigated the effect of a walnut-enriched diet on brain and liver oxylipin profile to get a general understanding of whether there would be a shift to less potent inflammatory mediators. Neuroinflammation is a chronic stressor that occurs during the ageing process (Neves and Sousa-Victor 2019; Cole et al. 2009; Hunt et al. 2019; Kim et al. 2016) and especially n3-PUFA are generally considered to attenuate the inflammatory response by increasing the production of less potent inflammatory mediators or anti-inflammatory metabolites altogether (Ostermann and Schebb 2017).

As it is already well documented in the literature, we are also able to report a significant increase of oxylipins derived from diet specific PUFA in the intervention groups (Nuernberg et al. 2011; Valencak and Ruf 2011). However, we found that the oxylipin profile differed in both tissues. This is in agreement with Naoe et al. (Naoe et al. 2019), who found similar results in liver and brain. In the liver, oxylipins of LA, ALA, EPA and DHA are increased compared to both groups adhering to the control diet, suggesting a successful modulation of the liver’s oxylipin profile. Similar, but smaller changes can be seen of the brain. On one hand walnut-based oxylipins were generally < LLOQ in control groups while being detectable in both intervention groups allowing only a qualitative analysis. On the other hand this confirmed that the brain was affected by the diet as well. Our data suggest that LA, ALA as well as EPA-derived oxylipin levels in the brain were all affected by the interventions, while only OH-PUFAs of ARA seemed to be reduced following walnut feeding and DHA-derived oxylipins were virtually identical with control groups. This is in agreement with our previous research and a study from Ferdouse et al. who also found a general increase in n3-PUFA derived oxylipins, but little to no changes in ARA-derived oxylipins (Ostermann et al. 2017a; Ferdouse et al. 2019) in the brain of rats. Furthermore, oxylipins derived from DHA, tend to be increased only in the liver, but not the brain. However, reports have already shown that production of DHA from ALA is limited (Demar et al. 2005). This is in line with our previous work and Ferdouse et al. who found DHA oxylipins levels to show the smallest changes if fed with either ALA, EPA or even DHA enriched diets (Ferdouse et al. 2019). This and the small changes to ARA-derived oxylipins seems to be specific to the brain, however, as we found the liver to be more susceptible to the diets components. This is generally in line with other studies reporting a stronger dietary modulation of the oxylipin profile in liver, kidney or adipose tissue (Leng et al. 2017,2018; Mendonça et al. 2018) of rats fed with LA, ALA, EPA or DHA diets. Accordingly, these studies also reported of significant changes to the n6/n3 ratio in liver or kidney, which we also observed for the liver, but not for the brain.

The Wal group showed only small trends to reduce prostanoid levels of 6-keto-PGF1α, PGF2α and TxB_2_ in the brain compared to the oldCon group, putting them more in line of those from youCon mice. However, additional physical enrichment of the WalEE mice could significantly enhance this effect. Amtul et al. found that increased PGE_2_, PGF2α and TxB_2_ drastically increased Aβ peptide concentrations in in vivo and in vitro models of AD (Amtul et al. 2012). Therefore, it might be possible that a walnut-enriched diet in combination with physical activity might provide a benefit against one of AD’s prominent pathologies. In the brain, PGD_3_, which is derived from EPA and formed in the COX pathway, is present in small amounts of several mice from both Wal and WalEE groups but was absent in all youCon and oldCon mice. This oxylipin is a less potent inflammatory mediator (Ostermann and Schebb 2017) compared to pro-inflammatory PGD_2_ which is formed in the same pathway originating from ARA. While Wal mice showed no changes in PGD_2_ levels, WalEE mice tended to show lower PGD_2_ and significantly lower TxB_2_ levels. Leng et al. found similar results and reported decreased PGD_2_ an TxB_2_ levels in the livers of rats accompanied by increased production of PGD_3_ and TxB_3_ (Leng et al. 2018). Nevertheless, here, their production was not detected in all intervention mice and was minimal compared to the high ARA-derived prostanoids. Still, their presence in the Wal and WalEE mice hints at a potential modulation towards less potent inflammatory mediators.

As Walnuts also contain high amounts of LA, the increases in hydroxy octadecadienoic acids (HODE) were also expected and suggest a modulation of the LOX products. Our recent work exploring the LOX enzyme affinity towards DHA supports this shift (Kutzner et al. 2019). As walnuts were the only source of ALA, its hydroxy-metabolites, i.e. hydroxy octadecatrienoic acids (HOTrE) were slightly increased in the intervention groups of the brain but more drastically in the liver, while being barely detectable in the control groups.

ALA-based epoxy octadecadienoic acids (EpODEs) were mostly not detectable in the brain, which again supports the generally lower effect of the diet on the brain and makes it difficult to determine the diets effect on the CYP pathway. However, looking at the liver increased CYP products could be found for LA, ALA as well as several Ep-PUFA of DHA and 17(18)-EpETE derived from EPA. Nevertheless, this modulation had no apparent effect on the ARA-derived CYP products in either group or tissue. Of all EpODEs, 15(16)-EpODE was the only oxylipin detectable in both intervention groups in the brain and was strongly increased in the liver and brain. DiHODEs, the hydrolysis products of EpODEs, which can be formed via sEH were increased in the same manner. In the liver, both intervention groups showed increased levels of epoxy octadecenoic acids (EpOME) twofold and their dihydroxy-metabolites by two–threefold. The ratio of Ep-PUFA/DiOH-PUFA implies a rather stable sEH activity, which further suggest that feeding of walnuts, rich in ALA and LA, did not affect sEH in the same way as supplementation with EPA and DHA did in our previous work (Ostermann and Schebb 2017).

As inflammation is an increasing factor during the ageing process, our data show a shift in the oxylipin profile of both brain and liver in line with the dietary supplementation. This shift is characterized by increased production of n3-PUFA oxylipins which are generally considered to be beneficial towards inflammation. Nevertheless, the production of n3-PUFA oxylipins in the brain was smaller compared to the liver again suggesting that possible effects of the n3-PUFA were very limited and effects on mitochondrial function might have been similarly small. Although walnuts tend to induce promising changes, more pronounced effects might be possible if supplementation of n3-PUFA is directly done with EPA and DHA. As we did not look at specific regions of the brain to evaluate the oxylipin profiles or the effect on mitochondrial function, unaffected regions of the brain might mask beneficial effects.

Looking at the expression of the inflammatory marker IL1β in brain and liver tissue, IL1β levels of either intervention very virtually identical compared to youCon in the liver. Aged control mice on the other hand showed a greater variability and tended to lean towards elevated IL1β expression. In the brain, the expression of IL1β was virtually identical across all groups. A recent study by Mejias et al. found that IL1b was increased in aged mice and rats (Mejias et al. 2018; Mawhinney et al. 2011). Lynch et al., as well as Minogue et al. indeed found that EPA reduced hippocampal IL1β expression in rats (Lynch et al. 2007; Minogue et al. 2007). Similarly to Moon et al., who reported a reduced IL1β expression and PGE2 levels in BV2 microglia upon EPA treatment, the walnut diet showed similar trends in the liver for both analytes. Nevertheless, it is uncertain whether the missing change in expression actually has an effect on the protein level. Future studies have to show whether the transcription data also lead to changes at the translation level. Furthermore, Poulose et al. also only found an effect in both striatum and hippocampus, when rats were fed with a 9% walnut-enriched diet (Poulose et al. 2013).

In conclusion, we observed a modulation of the oxylipin profile by a 6% walnut-enriched diet alone and combined with physical enrichment in mice. These effects might lead to better cognitive and motor functions in mice as seen in our results for Rotarod and Y-Maze. Effects of the interventions could not be linked to an enhanced mitochondrial function and gene expression of markers related to neurogenesis were not affected either. This, however, might be explained with the fact that this study did not investigate specific regions of the brain, but a broad selection of different regions in which unaffected areas might mask small beneficial effects in mitochondria. Furthermore, since results for gene expression do not indicate altered neuronal function, results from Y-maze alternation test should be verified by comparable tests. Results from a recent clinical trial, in which subjects adhered to a walnut-enriched diet for 2 years, did not show changes in cognition of healthy elderly patients (Sala-Vila et al. 2020). Future studies should take an in-depth look at different brain regions to isolated specific walnut-related effects. Here, we were able to show that a walnut-enriched diet affects cognition in aged NMRI mice and benefits their fatty acid composition to potentially attenuate age-related neuroinflammation in the ageing brain and body. Therefore, walnut-based nutrition might be a promising target for healthy ageing, but still needs to be further investigated.

## Supplementary Information

Below is the link to the electronic supplementary material.

Supplementary file1 (docx 222 KB)

## Data Availability

The dataset generated during this study is available from the corresponding author upon reasonable request.

## Abbreviations

Aβ: Amyloid-beta peptide
AD: Alzheimer’s Disease
ADP: Adenosine diphosphate
ALA: α-Linolenic acid, C18:3n3
ARA: Arachidonic acid, C20:4n6
ATP: Adenosine triphosphate
COX: Cyclooxygenase
CYP: Cytochrome P450 monooxygenase
DBC: Dissociated brain cells
DGLA: Dihomo-γ-linolenic acid, C20:3n6
DHA: Docosahexaenoic acid, C22:6n3
DiHEDE: Dihydroxy eicosadienoic acid
DiHETE: Dihydroxy eicosatetraenoic acid
DiHETrE: Dihydroxy eicosatrienoic acid
DiHDPE: Dihydroxy docosapentaenoic acid
DiHODE: Dihydroxy octadecadienoic acid
DiHOME: Dihydroxy octadecenoic acid
DiOH-PUFA: Dihydroxy polyunsaturated fatty acid
EPA: Eicosapentaenoic acid, C20:5n3
EpDPE: Epoxy docosapentaenoic acid
EpEDE: Epoxy eicosadienoic acid
EpETE: Epoxy eicosatetraenoic acid
EpETrE: Epoxy eicosatrienoic acid
EpODE: Epoxy octadecadienoic acid
EpOME: Epoxy octadecenoic acid
Ep-PUFA: Epoxy polyunsaturated fatty acid
ETC: Electron transfer chain
GC/MS: Gas chromatography/mass spectrometry
HDHA: Hydroxy docosahexaenoic acid
HEPE: Hydroxy eicosapentaenoic acid
HETE: Hydroxy eicosatetraenoic acid
HETrE: Hydroxy eicosatrienoic acid
HODE: Hydroxy octadecadienoic acid
HOTrE: Hydroxy octadecatrienoic acid
LA: Linoleic acid, C18:2n6
LLOQ: Lower limit of quantification
LN2: Liquid nitrogen
LOD: Limit of detection
LOX: Lipoxygenase
MiR05: Mitochondrial respiration medium
MMP: Mitochondrial membrane potential
n3-PUFA: Omega-3 polyunsaturated fatty acid
n6-PUFA: Omega-6 polyunsaturated fatty acid
NMRI: Naval Medical Research Institute
NO: Nitric oxide
OH-PUFA: Hydroxy-polyunsaturated fatty acid
oldCon: Old control group
OXPHOS: Oxidative phosphorylation
PG: Prostaglandins
PI: Protease inhibitor
PUFA: Polyunsaturated fatty acid
qRT-PCR: Quantitative real-time polymerase chain reaction
R123: Rhodamine 123
ROS: Reactive oxygen species
RP-LC-ESI(-)-MS/MS: Reverse-phase liquid chromatography negative electrospray ionization tandem mass spectrometry
sEH: Soluble epoxide hydrolase
SNP: Sodium nitroprusside
Tx: Thromboxanes
Wal: Walnut-only group
WalEE: Walnut + enriched environment group
youCon: Young control group
