# Supplementary material for: A Walnut Diet in Combination with Enriched Environment Improves Cognitive Function and Affects Lipid Metabolites in Brain and Liver of Aged NMRI Mice
Source: Neuromolecular Med. 2020 Dec 26;23(1):140–60. doi: 10.1007/s12017-020-08639-7 (PMC7929966; doi:10.1007/s12017-020-08639-7)
Supplement: Supplementary file 1 — Supplementary file1 (docx 222 KB) [file 12017_2020_8639_MOESM1_ESM.docx]

Table of Supplementary Figures and Tables

[*Supplementary Fig. 1:* 2](#_Toc54082292)

[*Supplementary Table 1:* 3](#_Toc54082293)

[*Supplementary Table 2:* 4](#_Toc54082294)

[*Supplementary Table 3:* 5](#_Toc54082295)

[*Supplementary Table 4:* 6](#_Toc54082296)

[*Supplementary Table 5:* 8](#_Toc54082297)

[*Supplementary Table 6:* 9](#_Toc54082298)

[*Supplementary Table 7:* 10](#_Toc54082299)

[*Supplementary Table 8:* 12](#_Toc54082300)

[*Supplementary Table 9:* 12](#_Toc54082301)

[*Supplementary Table 10:* 13](#_Toc54082302)


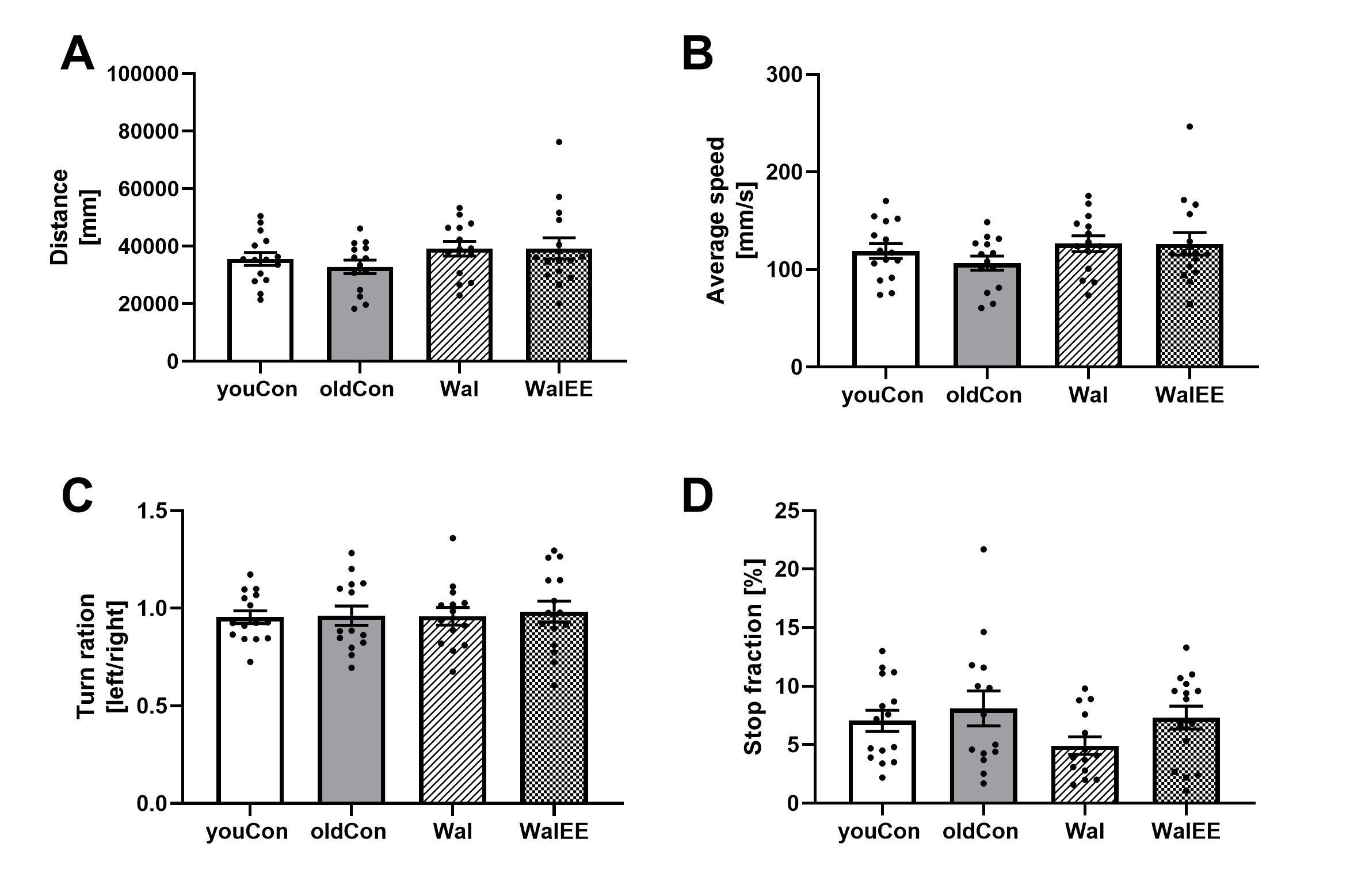


# *Supplementary Fig. 1:*

*Open Field results. Mice were placed in the arena for 5 minutes and their movement was recorded. A: Total distance travelled during a span of 5 minutes. B: Average speed of each group during the experiment. C: Ratio of the number of left and right turns in each group. D: Fraction of the time mice spent without moving in the open field area. Data is displayed as mean ± SEM; N = 14-15. To test significance an one-way ANOVA with posterior Dunnett’s posthoc test, comparing all groups with oldCon, was applied. oldCon = old Control; youCon = Young control; Wal = Walnut group; WalEE = Walnut+Enriched Environment group. Parameters for statistical testing can be found in the specific supplementary table.*

# *Supplementary Table 1:*

*Statistical parameters of behavioural testing. Significance was tested using an one-way ANOVA with Dunnett’s posthoc test, compairing all groups with oldCon. oldCon = old control, youCon = young control, Wal = Walnut group, WalEE = Walnut+Enriched Environment group. Given are the F and p values for the ANOVA as well as the specific p values for each comparison. A: Parameters for Y-Maze alternation test (Figure 2A-C); B: Parameters for Rotarod test (Figure 2D); C: Parameters for Passive Avoidance test (Figure 2E); D: Parameters for Open Field tests (Supplementary Figure 1).*

| **A)**  **Y-Maze alternation test** | **Number of entries**  **(F = 3.919) (p = 0.0150)** | **Number of alternations**  **(F = 6.796) (p = 0.0006)** | **Alternation rate**  **(F = 6.585) (p = 0.0007)** |
| --- | --- | --- | --- |
| oldCon vs. Wal | p = 0.0237 | p = 0.0157 | p = 0.0207 |
| oldCon vs. WalEE | p = 0.0090 | p = 0.0007 | p = 0.0421 |
| oldCon vs. youCon | p = 0.0842 | p = 0.0009 | p = 0.0002 |

| **B)**  **Rotarod test** | **Time to fall**  **(F = 10.78) (p < 0.0001)** |
| --- | --- |
| oldCon vs. Wal | p = 0.9999 |
| oldCon vs. WalEE | p = 0.0381 |
| oldCon vs. youCon | p < 0.0001 |

| **C)**  **Passive Avoidance** | **Time until entering (Day 1) (F = 0.9425) (p = 0.4269)** | **Time until entering (Day 2) (F = 1.527) (p = 0.2228)** |
| --- | --- | --- |
| oldCon vs. Wal | p = 0.5172 | p = 0.9716 |
| oldCon vs. WalEE | p = 0.9177 | p = 0.1507 |
| oldCon vs. youCon | p = 0.2956 | p = 0.9930 |

| **D)**  **Open Field** | **Distance**  **(F = 1.239)**  **(p = 0.3047)** | **Speed**  **(F = 1.083)**  **(p = 0.3641)** | **Turn rate**  **(F = 0.0787)**  **(p = 0.9713)** | **Stop fraction**  **(F = 1.599)**  **(p = 0.2002)** |
| --- | --- | --- | --- | --- |
| oldCon vs. Wal | p = 0.2684 | p = 0.2861 | p > 0.9999 | p = 0.1041 |
| oldCon vs. WalEE | p = 0.2454 | p = 0.2781 | p = 0.9781 | p = 0.9170 |
| oldCon vs. youCon | p = 0.8195 | p = 0.6334 | p = 0.9986 | p = 0.8212 |

# *Supplementary Table 2:*

*Statistical parameters of mitochondrial function. Significance was tested using an one-Way ANOVA with Dunnett’s posthoc test, compairing all groups with oldCon. oldCon = old control, youCon = young control, Wal = Walnut group, WalEE = Walnut+Enriched Environment group. Given are the F and p values for the ANOVA as well as the specific p values for each comparison. A: Parameters for respiration experiments (Figure 3A); B: Parameters for MMP (R123 fluorescence measurements) (Figure 3B&C); C: Parameters for ATP level determination (Figure 3D&E); D: Parameters for citrate synthase activity (Figure 3F).*

| **A)**  **Respiration** | **oldCon vs. Wal** | **oldCon vs. WalEE** | **oldCon vs. youCon** |
| --- | --- | --- | --- |
| CI (L)  (F = 0.04194) (P = 0.9884) | p = 0.9890 | p > 0.9999 | p = 0.9855 |
| CI (P)  (F = 0.4959) (P = 0.6873) | p = 0.9769 | p = 0.8754 | p = 0.8237 |
| CI & C2 (P)  (F = 0.6205) (P = 0.6062) | p = 0.8178 | p = 0.8282 | p = 0.9541 |
| Cytochrome C  (F = 1.674) (P = 0.1884) | p = 0.9269 | p = 0.1026 | p = 0.6640 |
| CI & C2 (E)  (F = 0.1156) (P = 0.9504) | p = 0.9962 | p = 0.9139 | p > 0.9999 |
| CII (E)  (F = 0.1388) (P = 0.9362) | p = 0.9997 | p = 0.9777 | p = 0.9776 |
| CII (L)  (F = 0.1825) (P = 0.9076) | p = 0.9979 | p = 0.9970 | p = 0.9047 |
| CIV (E)  (F = 0.2483) (P = 0.8620) | p = 0.9313 | p = 0.9385 | p = 0.9911 |

| **B)**  **MMP** | **oldCon vs. Wal** | **oldCon vs. WalEE** | **oldCon vs. youCon** |
| --- | --- | --- | --- |
| (F = 0.1243) (p = 0.9453) | p = 0.9993 | p = 0.8991 | p = 0.9986 |
| **MMP + 0.5 mM SNP** |  |  |  |
| (F = 3.061) (p = 0.0236) | p = 0.9997 | p = 0.9958 | p = 0.9450 |

| **C)**  **ATP** | **oldCon vs. Wal** | **oldCon vs. WalEE** | **oldCon vs. youCon** |
| --- | --- | --- | --- |
| (F = 0.7398) (p = 0.5330) | p = 0.9515 | p = 0.8905 | p = 0.3428 |
| **ATP + 0.5 mM SNP** |  |  |  |
| (F = 0.2752) (p = 0.8430) | p = 0.9691 | p > 0.9999 | p = 0.7788 |

| **D)**  **Citratsynthase actvity** | **oldCon vs. Wal** | **oldCon vs. WalEE** | **oldCon vs. youCon** |
| --- | --- | --- | --- |
| (F = 0.9843) (p = 0.4110) | p = 0.4822 | p = 0.9594 | p = 0.9035 |

# *Supplementary Table 3:*

*Statistical parameters of gene expression in brain and liver. Significance was tested using an one-Way ANOVA with Dunnett’s posthoc test, compairing all groups with oldCon. oldCon = old control, youCon = young control, Wal = Walnut group, WalEE = Walnut+Enriched Environment group. Given are the F and p values for the ANOVA as well as the specific p values for each comparison. A: Parameters for gene expression in the brain (Table 4); B: Parameters for gene expression in the liver (Table 7).*

| **A)**  **Gene expression in BRAIN** | **oldCon vs. Wal** | **oldCon vs. WalEE** | **oldCon vs. youCon** |
| --- | --- | --- | --- |
| Synaptophysin  (F = 2.340) (p = 0.0897) | p = 0.9999 | p = 0.6681 | p = 0.0776 |
| BDNF  (F = 2.939) (p = 0.0470) | p > 0.9999 | p = 0.9817 | p = 0.0432 |
| NGF  (F = 2.191) (p = 0.1060) | p = 0.9993 | p = 0.8209 | p = 0.0929 |
| Gap43  (F = 0.4783) (p = 0.7012) | p = 0.9343 | p = 0.8388 | p = 0.9558 |
| Keap1  (F = 1.354) (p = 0.2745) | p = 0.9153 | p = 0.5463 | p = 0.1514 |
| NRF2  (F = 1.863) (p = 0.1551) | p = 0.9705 | p = 0.8109 | p = 0.1766 |
| CREB1  (F = 7.013) (p = 0.0012) | p = 0.3317 | p = 0.8025 | p = 0.0149 |
| IL1β  (F = 1.215) (p = 0.3190) | p > 0.9999 | p = 0.2392 | p = 0.9434 |

| **B)**  **Gene expression in LIVER** | **oldCon vs. Wal** | **oldCon vs. WalEE** | **oldCon vs. youCon** |
| --- | --- | --- | --- |
| Keap1  (F = 2.303) (p = 0.0892) | p = 0.0395 | p = 0.1625 | p = 0.5165 |
| NRF2  (F = 4.267) (p = 0.0093) | p = 0.0709 | p = 0.1317 | p = 0.8532 |
| CREB1  (F = 1.025) (p = 0.3907) | p = 0.9994 | p = 0.5212 | p = 0.4749 |
| IL1b  (F = 2.024) (p = 0.1244) | p = 0.0932 | p = 0.1738 | p = 0.1074 |

# *Supplementary Table 4:*

*Statistical parameters of hydroxy- and epoxy-PUFA concentrations in liver and brain (Table 5) of all groups compared to oldCon. Data was statistically compared via one-Way ANOVA with Dunnett’s post-hoc test and compared to oldCon. A&B: Parameters for hydroxy- and epoxy-PUFA levels in the brain (Table 5A); C&D: Parameters for hydroxy- and epoxy-PUFA levels in the liver (Table 5B).*

| **A)**  **BRAIN**  **Hydroxy-PUFA** | **oldCon vs youCon** | **oldCon vs Wal** | **oldCon vs WalEE** |
| --- | --- | --- | --- |
| LA  (F = 7.833)  (p = 0.0002) | p = 0.9761 | p = 0.0025 | p = 0.0113 |
| ALA | OldCon < LLOQ | | |
| ARA  (F = 7.381)  (p = 0.0003) | p = 0.0148 | p = 0.0014 | p = 0.0002 |
| EPA  (F = 2.714)  (p = 0.0544) | p = 0.4561 | p = 0.8987 | p = 0.2613 |
| DHA  (F = 1.187)  (p = 0.3232) | p = 0.8545 | p = 0.2247 | p = 0.3537 |

| **B)**  **BRAIN**  **Epoxy-PUFA** | **oldCon vs youCon** | **oldCon vs Wal** | **oldCon vs WalEE** |
| --- | --- | --- | --- |
| LA | OldCon < LLOQ | | |
| ALA | OldCon < LLOQ | | |
| ARA  (F = 4.480)  (p = 0.0072) | p = 0.0439 | p = 0.9262 | p = 0.0097 |
| EPA | OldCon < LLOQ | | |
| DHA  (F = 4.081)  (p = 0.0113) | p = 0.2050 | p = 0.6511 | p = 0.1171 |

| **C)**  **LIVER**  **Hydroxy-PUFA** | **oldCon vs youCon** | **oldCon vs Wal** | **oldCon vs WalEE** |
| --- | --- | --- | --- |
| LA  (F = 20.74)  (p < 0.0001) | p = 0.9987 | p < 0.0001 | p < 0.0001 |
| ALA  (F = 23.74)  (p < 0.0001) | p = 0.9999 | p < 0.0001 | p < 0.0001 |
| ARA  (F = 1.938)  (p = 0.1349) | p = 0.1751 | p = 0.6246 | p = 0.9759 |
| EPA  (F = 38.35)  (p < 0.0001) | p > 0.9999 | p < 0.0001 | p < 0.0001 |
| DHA  (F = 28.73)  (p < 0.0001) | p = 0.9922 | p < 0.0001 | p < 0.0001 |

*Supplementary Table 4 cont:*

| **D)**  **LIVER**  **Epoxy-PUFA** | **oldCon vs youCon** | **oldCon vs Wal** | **oldCon vs WalEE** |
| --- | --- | --- | --- |
| LA  (F = 23.59)  (p < 0.0001) | p = 0.9988 | p = 0.0003 | p < 0.0001 |
| ALA | oldCon < LLOQ | | |
| ARA  (F = 2.315)  (p = 0.0858) | p = 0.6860 | p = 0.5710 | p = 0.4498 |
| EPA | oldCon < LLOQ | | |
| DHA  (F = 34.54)  (p < 0.0001) | p = 0.9980 | p < 0.0001 | p < 0.0001 |

# *Supplementary Table 5:*

*Significance levels of selected oxylipins shown in Heatmap of BRAIN (Figure 4A). Significance was tested via one-way ANOVA with Dunnett’s posthoc test comparing all groups with oldCon. If <LLOQ is displayed, statistical significance could not be calculated, as analyte could not be detected in the experiment. Significances are displayed as *p<0.05, **p<0.01, ***p<0.001, **** p<0.0001.*

| **Brain** |  | **oldCon vs youCon** | **oldCon vs Wal** | **oldCon vs WalEE** |
| --- | --- | --- | --- | --- |
| LA | **9-HODE**  (F = 8.374)  (p = 0.0001) | Ns  p = 0.9716 | **  p = 0.0062 | **  p < 0.0024 |
|  | **13-HODE**  (F = 6.749)  (p = 0.0006) | Ns  p = 0.9853 | **  p = 0.0045 | *  p = 0.0219 |
|  | **9(10)-EpOME** | oldCon < LLOQ | | |
|  | **12(13)-EpOME** | oldCon < LLOQ | | |
|  | **9,10-DiHOME** | oldCon < LLOQ | | |
|  | **12,13-DiHOME** | oldCon < LLOQ | | |
| ALA | **13-HOTrE** | oldCon < LLOQ | | |
|  | **15(16)-EpODE** | oldCon < LLOQ | | |
|  | **9,10-DiHODE**  (F = 5.174)  (p = 0.0032) | Ns  p = 0.8635 | Ns  p = 0.0615 | **  p = 0.0022 |
|  | **12,13-DiHODE** | oldCon < LLOQ | | |
|  | **15,16-DiHODE** | oldCon < LLOQ | | |
| ARA | **5-HETE**  (F = 3.644)  (p = 0.0181) | Ns  p = 0.5576 | **  p = 0.0091 | Ns  p = 0.0626 |
|  | **9-HETE**  (F = 0.9765)  (p = 0.4105) | Ns  p = 0.9331 | Ns  p = 0.6158 | Ns  p = 0.9326 |
|  | **12-HETE**  (F = 7.851)  (p = 0.002) | *  p = 0.0150 | ***  p = 0.0002 | ***  p = 0.0005 |
|  | **15-HETE**  (F = 4.455)  (p = 0.0071) | Ns  p = 0.6125 | Ns  p = 0.1059 | **  p = 0.0026 |
|  | **20-HETE**  (F = 0.6068)  (p = 0.6135) | Ns  p = 0.9024 | Ns  p = 0.7936 | Ns  p = 0.9971 |
|  | **14(15)-EpETrE**  (F = 4.496)  (p = 0.0071) | *  p = 0.0427 | Ns  p = 0.8829 | **  p = 0.0082 |
| EPA | **5-HEPE** | oldCon < LLOQ | | |
|  | **9-HEPE** | oldCon < LLOQ | | |
|  | **12-HEPE**  (F = 1.922)  (p = 0.1375) | Ns  p = 0.3518 | Ns  p = 0.9988 | Ns  p = 0.7549 |
|  | **15-HEPE** | oldCon < LLOQ | | |
|  | **18-HEPE**  (F = 45.67)  (p < 0.0001) | youCon < LLOQ | ****  p < 0.0001 | ****  p < 0.0001 |
|  | **17(18)-EpETE** | oldCon < LLOQ | | |
| DHA | **4-HDHA**  (F = 1.329)  (p = 0.2744) | Ns  p = 0.4467 | Ns  p = 0.9794 | Ns  p = 0.2068 |
|  | **7-HDHA**  (F = 0.1815)  (p < 0.0001) | Ns  p = 0.9801 | Ns  p = 0.9963 | Ns  p = 0.9266 |
|  | **14-HDHA**  (F = 4.769)  (p = 0.0052) | *  p = 0.0353 | **  p = 0.0033 | *  p = 0.0146 |
|  | **17-HDHA**  (F = 0.5295)  (p = 0.6639) | Ns  p = 0.6228 | Ns  p = 0.9138 | Ns  p = 0.9990 |
|  | **19(20)-EpDPE**  (F = 4.102)  (p = 0.0113) | Ns  p = 0.1611 | Ns  p = 0.7447 | Ns  p = 0.0979 |

# *Supplementary Table 6:*

*Significance levels of selected oxylipins shown in Heatmap of LIVER (Figure 4B). Significance was tested via one-way ANOVA with Dunnett’s posthoc test comparing all groups to oldCon. If <LLOQ is displayed, statistical significance could not be calculated, as analyte could not be detected in the experiment. For 20-HETE (marked with *) both youCon and Wal were <LLOQ. In this case significance was calculated using an unpaired-t-test between oldCon and WalEE. Significances are displayed as *p<0.05, **p<0.01, ***p<0.001, **** p<0.0001.*

| **Liver** |  | **oldCon vs youCon** | **oldCon vs Wal** | **oldCon vs WalEE** |
| --- | --- | --- | --- | --- |
| LA | **9-HODE**  (F = 21.44)  (p < 0.0001) | Ns  p = 0.9999 | ***  p = 0.0006 | ****  p < 0.0001 |
|  | **13-HODE**  (F = 13.38)  (p < 0.0001) | Ns  p = 0.9990 | ****  p < 0.0001 | ***  p = 0.0001 |
|  | **9(10)-EpOME**  (F = 20.85)  (p < 0.0001) | Ns  p = 0.9989 | **  p = 0.0019 | ****  p < 0.0001 |
|  | **12(13)-EpOME**  (F = 26.19)  (p < 0.0001) | Ns  p = 0.9987 | ****  p < 0.0001 | ****  p < 0.0001 |
|  | **9,10-DiHOME**  (F = 23.64)  (p < 0.0001) | Ns  p > 0.9999 | **  p = 0.0031 | ****  p < 0.0001 |
|  | **12,13-DiHOME**  (F = 40.81)  (p < 0.0001) | Ns  p = 0.9622 | ****  p < 0.0001 | ****  p < 0.0001 |
| ALA | **13-HOTrE** | oldCon < LLOQ | | |
|  | **15(16)-EpODE** | oldCon < LLOQ | | |
|  | **9,10-DiHODE**  (F = 31.92)  (p < 0.0001) | Ns  p > 0.9999 | ****  p < 0.0001 | ****  p < 0.0001 |
|  | **12,13-DiHODE** | oldCon < LLOQ | | |
|  | **15,16-DiHODE**  (F = 60.37)  (p < 0.0001) | Ns  p > 0.9999 | ****  p < 0.0001 | ****  p < 0.0001 |
| ARA | **5-HETE**  (F = 2.438)  (p = 0.0740) | Ns  p = 0.3860 | Ns  p = 0.9923 | Ns  p = 0.4143 |
|  | **9-HETE**  (F = 2.924)  (p = 0.0416) | Ns  p = 0.4962 | Ns  p = 0.9284 | Ns  p = 0.2122 |
|  | **12-HETE**  (F = 0.3165)  (p = 0.8134) | Ns  p > 0.9999 | Ns  p = 0.8222 | Ns  p = 0.8323 |
|  | **15-HETE**  (F = 1.147)  (p = 0.3382) | Ns  p = 0.3091 | Ns  p = 0.3172 | Ns  p = 0.9320 |
|  | **20-HETE^+^** | youCon < LLOQ | Wal < LLOQ | Ns  p = 0.2385 |
|  | **14(15)-EpETrE**  (F = 3.526)  (p = 0.0206) | Ns  p = 0.3799 | Ns  p = 0.8486 | Ns  p = 0.2136 |
| EPA | **5-HEPE**  (F = 47.31)  (p < 0.0001) | Ns  p = 0.9999 | ****  p < 0.0001 | ****  p < 0.0001 |
|  | **9-HEPE** | oldCon < LLOQ | | |
|  | **12-HEPE**  (F = 15.49)  (p < 0.0001) | youCon < LLOQ | ***  p = 0.0003 | ****  p < 0.0001 |
|  | **15-HEPE** | oldCon < LLOQ | | |
|  | **18-HEPE**  (F = 25.99)  (p < 0.0001) | Ns  p = 0.9977 | ****  p < 0.0001 | ****  p < 0.0001 |
|  | **17(18)-EpETE** | oldCon < LLOQ | | |
| DHA | **4-HDHA**  (F = 18.98)  (p < 0.0001) | Ns  p = 0.9991 | ****  p < 0.0001 | ****  p < 0.0001 |
|  | **7-HDHA**  (F = 27.96)  (p < 0.0001) | Ns  p > 0.9999 | ****  p < 0.0001 | ****  p < 0.0001 |
|  | **14-HDHA**  (F = 15.28)  (p < 0.0001) | Ns  p = 0.9994 | ****  p < 0.0001 | ****  p < 0.0001 |
|  | **17-HDHA** | oldCon < LLOQ | | |
|  | **19(20)-EpDPE**  (F = 27.89)  (p < 0.0001) | Ns  p = 0.9999 | ****  p < 0.0001 | ****  p < 0.0001 |

# *Supplementary Table 7:*

*Statistical parameters of ARA derived prostaglandins in brain and liver (Figure 5). Significance was tested via one-way ANOVA with Dunnett’s posthoc test comparing all groups to oldCon. A: Parameters for ARA derived prostanoids in the brain; B: Parameters for ARA derived prostanoids in the liver.*

| **A)**  **Brain** | **oldCon vs youCon** | **oldCon vs Wal** | **oldCon vs WalEE** |
| --- | --- | --- | --- |
| **PGD2**  (F = 1.086)  (p = 0.3628) | p = 0.6238 | p > 0.9999 | p = 0.3527 |
| **PGE2**  (F = 3.636)  (p = 0.0182) | p = 0.0447 | p = 0.9449 | p = 0.0320 |
| **6-keto-PGF1α**  (F = 2.637)  (p = 0.0589) | p = 0.9365 | p = 0.1551 | p = 0.0478 |
| **PGF2α**  (F = 5.949)  (p = 0.0014) | p = 0.3389 | p = 0.4428 | p = 0.0004 |
| **TxB2**  (F = 7.341)  (p = 0.0003) | p = 0.0013 | p = 0.4835 | p = 0.0008 |

| **B)**  **Liver** | **oldCon vs youCon** | **oldCon vs Wal** | **oldCon vs WalEE** |
| --- | --- | --- | --- |
| **PGD2**  (F = 4.765)  (p = 0.0055) | p = 0.1273 | p = 0.3149 | p = 0.3728 |
| **PGE2**  (F = 1.525)  (p = 0.2313) | youCon < LLOQ | p = 0.1702 | p = 0.8526 |
| **6-keto-PGF1α**  (F = 1.746)  (p = 0.1708) | p = 0.1312 | p = 0.4562 | p = 0.9979 |
| **PGF2α**  (F = 6.749)  (p = 0.0006) | youCon < LLOQ | p = 0.9677 | p = 0.0035 |
| **TxB2**  (F = 2.797)  (p = 0.0496) | p = 0.2499 | p = 0.4400 | p = 0.6557 |

# *Supplementary Table 8:*

*Statistical parameters for comparison of n6/n3 ratio in brain and liver (Table 6). Significance was tested via one-way ANOVA with Dunnett’s posthoc test comparing all groups to oldCon.*

| **n6/n3 ratio** | **oldCon vs youCon** | **oldCon vs Wal** | **oldCon vs WalEE** |
| --- | --- | --- | --- |
| Brain  (F = 1.563)  (p = 0.2087) | p = 0.6538 | p = 0.7980 | p = 0.0949 |
| Liver  (F = 65.87)  (p < 0.0001) | p = 0.626 | p < 0.0001 | p < 0.0001 |

# *Supplementary Table 9:*

*Mean number of feacal boli during the open field experiment. Data is displayed as mean ± SEM. Statistical significance in graph D was tested via One-Way ANOVA with Dunnett’s post-test comparing all groups with oldCon. Each group tested consisted of 15 female NMRI mice. Each group consisted of 15-17 mice.*

|  | **OldCon** | **YouCon** | **Wal** | **WalEE** |
| --- | --- | --- | --- | --- |
| Mean no. of feacal boli  (F = 0.2151) (p = 0.8856) | 3.0 ± 0.5  Ref. | 2.7 ± 0.4  n.s.  p = 0.9680 | 3.2 ± 0.5  n.s.  p = 0.9836 | 2.7 ± 0.6  n.s.  p = 0.9543 |

# *Supplementary Table 10:*

*Mean weight of mice during the whole feeding period. Data is displayed as mean ± SEM. Each group of old mice started with 22 twelve months old mice in week 0. At the end of the feeding period of 6 months only 17 oldCon mice, 16 Wal mice and 17 WalEE mice were still alive. Unless otherwise stated, death of mice occurred due to old age. Statistical testing was done using an one-way ANOVA with Dunnett’s posthoc test comparing Wal and WalEE with oldCon. YouCon was not included in statistical evaluation, as mice had not reached adulthood when they were included into the study. Their weight therefore still increased naturally.*

|  | **Mean weight [g]** | | | |
| --- | --- | --- | --- | --- |
|  | **OldCon** | **YouCon** | **Wal** | **WalEE** |
| Week 0 | 39.8 ± 1.4 |  | 40.8 ± 1.8 | 42.9 ± 1.6 |
| Week 1 | 41.1 ± 1.5 |  | 42.1 ± 2.3 | 40.1 ± 1.2 |
| Week 2 | 41.9 ± 1.8 |  | 44.2 ± 2.5 | 41.7 ± 1.4 |
| Week 3 | 42.1 ± 1.7 |  | 44.5 ± 2.8 | 42.1 ± 1.2 |
| Week 4 | 43.0 ± 1.8 |  | 44.1 ± 2.8 | 43.6 ± 1.5 |
| Week 6 | 42.9 ± 2.0 |  | 44.0 ± 2.8 | 42.3 ± 1.3 |
| Week 8 | 42.7 ± 2.0 |  | 43.3 ± 2.7 | 43.5 ± 1.6 |
| Week 10 | 43.2 ± 2.1 |  | 43.5 ± 3.2 | 45.5 ± 2.1 |
| Week 14 | 42.0 ± 2.2 | 16.47 ± 0.19 | 42.5 ± 3.0 | 42.5 ± 1.9 |
| Week 18 | 40.0 ± 1.9 | 24.7 ± 0.46 | 42.2 ± 2.9 | 45.0 ± 2.1 |
| Week 23 | 39.7 ± 1.9 | 29.0 ± 0.94 | 42.8 ± 2.47 | 45.7 ± 2.1 |
| Mean weight, total time  (F = 3.927)  (p = 0.0306) | 41.7 ± 0.40  Ref. |  | 43.1 ± 0.34  *  p = 0.0461 | 43.2 ± 0.51  *  p = 0.0343 |
